# Computing simplicial representatives of homotopy group elements

**DOI:** 10.1007/s41468-018-0021-5

**Published:** 2018-09-25

**Authors:** Marek Filakovský, Peter Franek, Uli Wagner, Stephan Zhechev

**Affiliations:** IST Austria, Am Campus 1, 3400 Klosterneuburg, Austria

**Keywords:** Computational homotopy theory, Effective Hurewicz Theorem, Whitehead tower, 68U05, 68W99, 68Q17, 55Q05, 55P99

## Abstract

A central problem of algebraic topology is to understand the *homotopy groups*$\pi _d(X)$ of a topological space *X*. For the computational version of the problem, it is well known that there is no algorithm to decide whether the *fundamental group*$\pi _1(X)$ of a given finite simplicial complex *X* is trivial. On the other hand, there are several algorithms that, given a finite simplicial complex *X* that is *simply connected* (i.e., with $\pi _1(X)$ trivial), compute the higher homotopy group $\pi _d(X)$ for any given $d\ge 2$. However, these algorithms come with a caveat: They compute the isomorphism type of $\pi _d(X)$, $d\ge 2$ as an *abstract* finitely generated abelian group given by generators and relations, but they work with very implicit representations of the elements of $\pi _d(X)$. Converting elements of this abstract group into explicit geometric maps from the *d*-dimensional sphere $S^d$ to *X* has been one of the main unsolved problems in the emerging field of computational homotopy theory. Here we present an algorithm that, given a simply connected space *X*, computes $\pi _d(X)$ and represents its elements as simplicial maps from a suitable triangulation of the *d*-sphere $S^d$ to *X*. For fixed *d*, the algorithm runs in time exponential in $\mathrm {size}(X)$, the number of simplices of *X*. Moreover, we prove that this is optimal: For every fixed $d\ge 2$, we construct a family of simply connected spaces *X* such that for any simplicial map representing a generator of $\pi _d(X)$, the size of the triangulation of $S^d$ on which the map is defined, is exponential in $\mathrm {size}(X)$.

## Introduction

One of the central concepts in topology are the *homotopy groups*$\pi _d(X)$ of a topological space *X*. Similar to the *homology groups*$H_d(X)$, the homotopy groups $\pi _d(X)$ provide a mathematically precise way of measuring the “*d*-dimensional holes” in *X*, but the latter are significantly more subtle and computationally much less tractable than the former. Understanding homotopy groups has been one of the main challenges propelling research in algebraic topology, with only partial results so far despite an enormous effort (see, e.g., Ravenel 2004; Kochman 1990); the amazing complexity of the problem is illustrated by the fact that even for the 2-dimensional sphere $S^2$, the higher homotopy groups $\pi _d(S^2)$ are nontrivial for infinitely many *d* and *known* only for a few dozen values of *d*.

For computational purposes, we consider spaces that have a combinatorial description as *simplicial sets* (or, alternatively, finite simplicial complexes) and maps between them as *simplicial maps*.

A fundamental computational result about homotopy groups is negative: There is no algorithm to decide whether the *fundamental group*$\pi _1(X)$ of a finite simplicial complex *X* is trivial, i.e., whether every continuous map from the circle $S^1$ to *X* can be continuously contracted to a point; this holds even if *X* is restricted to be 2-dimensional.^1^

On the other hand, given a space *X* that is *simply connected* (i.e., path connected and with $\pi _1(X)$ trivial) there are algorithms that compute the higher homotopy group $\pi _d(X)$, for every given $d \ge 2$. The first such algorithm was given by Brown (1957), and newer ones have been obtained as a part of general computational frameworks in algebraic topology; in particular, an algorithm based on the methods of Sergeraert (1994) and Rubio and Sergeraert (2002) was described by Real (1996).

More recently, Čadek et al. (2014b) proved that, for any fixed *d*, the homotopy group $\pi _d(X)$ of a given 1-connected finite simplicial set can be computed in polynomial time. On the negative side, computing $\pi _d(X)$ is #P-hard if *d* is part of the input (Anick 1989; Čadek et al. 2013b) (and, moreover, W[1]-hard with respect to the parameter *d*Matoušek 2014), even if *X* is restricted to be 4-dimensional. These results form part of a general effort to understand the computational complexity of topological questions concerning the classification of maps up to homotopy (Čadek et al. 2013a, b, 2014a; Filakovský and Vokřínek 2013) and related questions, such as the *embeddability problem* for simplicial complexes (a higher-dimensional analogue of graph planarity) (Matoušek et al. 2011, 2014; Čadek et al. 2017).

### Our results: representing homotopy classes by explicit maps

By definition, elements of $\pi _d(X)$ are equivalence classes of continuous maps from the *d*-dimensional sphere $S^d$ to *X*, with maps being considered equivalent (or lying in the same *homotopy class*) if they are *homotopic*, i.e. if they can be continuously deformed into one another (see Sect. 3 for more details).

The algorithms of Brown (1957) or Čadek et al. (2014b) mentioned above compute $\pi _d(X)$ as an abstract abelian group, in terms of generators and relations.^2^ However, they work with very implicit representations of the elements of $\pi _d(X)$.

On the other hand, assuming that *X* is finite, 0-reduced and $(d-1)$ connected, Berger (1991, 1995) presented an algorithm that computes generators of $\pi _d (X)$ as explicit simplicial maps.

Combining this algorithm with an algorithmic construction of the Whitehead tower, we managed to drop the condition on the connectivity and obtained the main result of this paper: an algorithm that, given an element $\alpha$ of $\pi _d(X)$, computes a suitable triangulation ${\varSigma }^d$ of the sphere $S^d$ and an explicit simplicial map ${\varSigma }^d \rightarrow X$ representing the given homotopy class $\alpha$.

Apart from the intrinsic importance of homotopy groups, we see this as a step towards the more general goal of computing explicit maps with specific topological properties; instances of this goal include computing explicit representatives of homotopy classes of maps between more general spaces *X* and *Y* (a problem raised in Čadek et al. 2014a) as well as *computing an explicit embedding* of a given simplicial complex into ${\mathbb {R}}^d$ (as opposed to *deciding embeddability*). Moreover, these questions are also closely related to *quantitative* questions in homotopy theory (Gromov 1999) and in the theory of embeddings (Freedman and Krushkal 2014). See Sect. 1.2 for a more detailed discussion of these questions.

Throughout this paper, we assume that the input *X* is *simply connected*, i.e., that it is connected and has trivial fundamental group $\pi _1(X)$. For the purpose of the exposition, we will assume that *X* is given as a 1-reduced simplicial set, encoded as a list of its nondegenerate simplices and boundary operators given via finite tables. We remark that the class of 1-reduced simplicial sets contains standard models of 1-connected topological spaces, such as spheres or complex projective spaces. A more general version of the theorem that also includes simply connected simplicial complexes is discussed in Sect. 4.

#### Theorem A

There exists an algorithm that, given $d\ge 2$ and a finite 1-reduced simplicial set *X*, computes a set of generators $g_1,\ldots , g_k$ of $\pi _d(X)$ as simplicial maps ${\varSigma }_j^d\rightarrow X$, for suitable triangulations ${\varSigma }_j^d$ of $S^d$, $j=1,\ldots ,k$.

For fixed *d*, the time complexity is exponential in the *size* (number of simplices) of *X*; more precisely, it is $O(2^{P(\mathrm {size}(X))})$ where $P=P_d$ is a polynomial depending only on *d*.

Any element of $\pi _d(X)$ can be expressed as a sum of generators, and expressing the sum of two explicit maps from spheres into *X* as another explicit map is a simple operation. Hence, the algorithm in Theorem A can convert *any* element of $\pi _d(X)$ into an explicit simplicial map.

Theorem A also has the following *quantitative* consequence: Fix some standard triangulation ${\varSigma }$ of the sphere $S^d$, e.g., as the boundary of a $d+1$-simplex. By the classical *Simplicial Approximation Theorem* (Hatcher 2001, 2.C), for any continuous map $f:S^d \rightarrow X$, there is a subdivision ${\varSigma }'$ of ${\varSigma }$ and a simplicial map $f':{\varSigma }'\rightarrow X$ that is homotopic to *f*. Theorem A implies that if *f* represents a generator of $\pi _d(X)$, then the size of ${\varSigma }'$ can be bounded by an exponential function of the number of simplices of *X*.

Furthermore, we can show that the exponential dependence on the number of simplices in *X* is inevitable:

#### Theorem B

Let $d\ge 2$ be fixed. Then there is an infinite family of *d*-dimensional 0-reduced 1-connected simplicial sets *X* such that for any simplicial map ${\varSigma }\rightarrow X$ representing a generator of $\pi _d(X)$, the triangulation ${\varSigma }$ of $S^d$ on which *f* is defined has size at least $2^{{\varOmega }(\mathrm {size}(X))}$. If $d\ge 3$, we may even assume that *X* are 1-reduced.

Consequently, any algorithm for computing simplicial representatives of the generators of $\pi _d(X)$ for 1-reduced simplicial set *X* has time complexity at least $2^{{\varOmega }(\mathrm {size}(X))}$.

In Sects. 4 and 5, we state and prove generalizations of Theorems A and B denoted as Theorems A.1 and B.1 . They remove the assumption that *X* is 1-reduced and replace it by a more flexible certificate of simply connectedness, allowing the input space *X* to be a more flexible simplicial set or simplicial complex.

This reduction from simplicial sets to simplicial complexes is achieved using a technical result we formulate later in the text as Lemma 6. The main ideas of this Lemma can be summarized as follows. For a finite simplicial complex $X^{sc}$ endowed with a certificate of 1-connectedness, we choose a spanning tree *T* and contract it into a point, creating a 0-reduced simplicial set $X=X^{sc}/T$. The certificate of 1-connectedness transfers to *X* and generalizes the 1-reduceness assumption in Theorem A. Once we compute a homotopy representative ${\varSigma }\rightarrow X$, we then convert it to an equivalent map $Sd({\varSigma })\rightarrow X^{sc}$ where *Sd* is a suitable subdivision functor, see Sect. 8 for details.

#### Source of the exponential

Let us briefly discuss the source of the exponential time complexity bound: Given the *X* as an input in Theorem A, the algorithm computes a set of generators of $\pi _d(X)$. These have an algebraic representation as elements of a simplicial group *G*. In particular, a generator $g \in G$ of $\pi _d$ has a form $g = \gamma _1 ^{\alpha _1}\cdots \gamma _n ^{\alpha _n}$, where the elements $\gamma _i$ are some agreed upon generators of *G*. The size of the exponents $\alpha _i$ is considered in a standard way (i.e. number of bits). All steps are polynomial up to this point.

The exponential blowup happens, when we assign a simplicial model of a sphere to $$g = \gamma _1 ^{\alpha _1}\cdots \gamma _n ^{\alpha _n}$$. The resulting sphere will contain $$\sim $$$$\sum _{i = 1} ^{n} |\alpha _i|$$ number of distinct *d*-simplices. This number can be large (even though its bit-size is polynomial). Hence, just outputting all these simplices could have exponential-time complexity in the input. In Theorem B, we show that this blowup really happens.

We remark that, in the boundary case of 1-reduced simplicial sets for $d=2$ (outside the scope of Theorem B), we don’t know whether the lower complexity bound is sub-exponential or not. However, we can show that the algorithm from Theorem A is optimal in that case as well, see a discussion in Sect. 5.

### Related and future work

#### Computational homotopy theory and applications

This paper falls into the broader area of *computational topology*, which has been a rapidly developing area (see, for instance, the textbooks Edelsbrunner and Harer 2010; Zomorodian 2005; Matveev 2007); more specifically, as mentioned above, this work forms part of a general effort to understand the computational complexity of problems in *homotopy theory*, both because of the intrinsic importance of these problems in topology and because of applications in other areas, e.g., to algorithmic questions regarding embeddability of simplicial complexes (Matoušek et al. 2011; Čadek et al. 2017), to questions in topological combinatorics (see, e.g., Mabillard and Wagner 2016), or to the robust satisfiability of equations (Franek and Krčál 2015).

A central theme in topology is to understand the set [*X*, *Y*] of all homotopy classes of maps from a space *X* to a space *Y*. In many cases of interest, this set carries additional structure, e.g., an abelian group structure, as in the case $\pi _d(X)=[S^d,X]$ of higher homotopy groups that are the focus of the present paper.

Homotopy-theoretic questions have been at the heart of the development of algebraic topology since the 1940’s. In the 1990s, three independent groups of researchers proposed general frameworks to make various more advanced methods of algebraic topology (such as spectral sequences) *effective* (algorithmic): Schön (1991), Smith (1998), and Sergeraert, Rubio, Dousson, Romero, and coworkers (e.g., Sergeraert 1994; Rubio and Sergeraert 2002, 2005; Romero et al. 2006; also see Rubio and Sergeraert 2012 for an exposition). These frameworks yielded general *computability* results for homotopy-theoretic questions (including new algorithms for the computation of higher homotopy groups Real 1996), and in the case of Sergeraert et al., also a *practical implementation* in form of the Kenzo software package (Heras et al. 2011).

Building on the framework of *objects with effective homology* by Sergeraert et al., in recent years a variety of new results in computational homotopy theory were obtained (Čadek et al. 2013b, 2014a, b, 2017; Krčál et al. 2013; Vokřínek 2017; Filakovský and Vokřínek 2013; Romero and Sergeraert 2012, 2016), including, in some cases, the first *polynomial-time algorithms*, by using a refined framework of *objects with polynomial-time homology* (Krčál et al. 2013; Čadek et al. 2014b) that allows for a computational complexity analysis. For an introduction to this area from a theoretical computer science perspective and an overview of some of these results, see, e.g., Čadek et al. (2013a) and the references therein.

#### Explicit maps

As mentioned above, the above algorithms often work with rather *implicit* representations of the homotopy classes in $\pi _d(X)$ (or, more generally, in [*X*, *Y*]) but does not yields explicit maps representing these homotopy classes.

For instance, the algorithm in Real (1996) computes $\pi _d(X)$ as the *homology group*$H_d(F)$ of an auxiliary space $F=F_d(X)$ constructed from *X* in such a way that $\pi _d(X)$ and $H_d(F)$ are isomorphic as groups.^3^

More recently, Romero and Sergeraert (2016) devised an algorithm that, given a 1-reduced (and hence simply connected) simplicial set *X* and $d\ge 2$, computes the homotopy group $\pi _d(X)$ as the homotopy group $\pi _d(K)$ of an auxiliary simplicial set *K* (a so-called *Kan completion* of *X*) with $\pi _d(X)\cong \pi _d(K)$. Moreover, given an element of this group, the algorithm can compute an explicit simplicial map ${\varSigma }^d \rightarrow K$ from a suitable triangulation of $S^d$ to *K* representing the given homotopy class. In this way, homotopy classes are represented by explicit maps, but as maps to the auxiliary space *K*, which is homotopy equivalent to but not homeomorphic to the given space *X*.

By contrast, our general goal is to is represent homotopy classes by maps into the given space; in the present paper, we treat, as an important first instance, the case $\pi _d(X)=[S^d,X]$.

#### Open problems and future work

Our next goal is to extend the results here to the setting of Čadek et al. (2014a), i.e., to represent, more generally, homotopy classes in [*X*, *Y*] by explicit simplicial maps from some suitable subdivision $X'$ to *Y* (under suitable assumptions that allow us to compute [*X*, *Y*]).^4^

In a subsequent step, we hope to generalize this further to the *equivariant* setting $[X,Y]_G$ of Čadek et al. (2017), in which a finite group *G* of symmetries acts on the spaces *X*, *Y* and all maps and homotopies are required to be *equivariant*, i.e., to preserve the symmetries.

As mentioned above, one motivation is the problem of algorithmically constructing embeddings of simplicial complexes into ${\mathbb {R}}^d$. Indeed, in a suitable range of dimensions ($d\ge \frac{3(k+1)}{2}$), the existence of an embedding of a finite *k*-dimensional simplicial complex *K* into ${\mathbb {R}}^d$ is equivalent to the existence of an ${\mathbb {Z}}_2$-equivariant map from an auxiliary complex ${\tilde{K}}$ (the deleted product) into the sphere $S^{d-1}$, by a classical theorem of Haefliger (1962) and Weber (1967). The proof of the Haefliger–Weber Theorem is, in principle, constructive, but in order to turn this construction into an algorithm to compute an embedding, one needs an explicit equivariant map into the sphere $S^{d-1}$.

#### Quantitative homotopy theory

Another motivation for representing homotopy classes by simplicial maps and complexity bounds for such algorithms is the connection to *quantitative questions* in homotopy theory (Gromov 1999; Ferry and Weinberger 2013) and in the theory of embeddings (Freedman and Krushkal 2014). Given a suitable measure of *complexity* for the maps in question, typical questions are: What is the relation between the complexity of a given null-homotopic map $f: X\rightarrow Y$ and the minimum complexity of a nullhomotopy witnessing this? What is the minimum complexity of an embedding of a simplicial complex *K* into ${\mathbb {R}}^d$? In quantitative homotopy theory, complexity is often quantified by assuming that the spaces are metric spaces and by considering Lipschitz constants (which are closely related to the sizes of the simplicial representatives of maps and homotopies Ferry and Weinberger 2013). For embeddings, the connection is even more direct: a typical measure is the smallest number of simplices in a subdivision $K'$ or *K* such that there exists a simplexwise linear-embedding $K' \hookrightarrow {\mathbb {R}}^d$.

### Structure of the paper

The remainder of the paper is structured as follows: In Sect. 2, we give a high-level description of the main ingredients of the algorithm from Theorem A. In Sect. 3, we review a number of necessary technical definitions regarding simplicial sets and the frameworks of effective and polynomial-time homology, in particular Kan’s simplicial version of loop spaces and polynomial-time loop contractions for infinite simplicial sets. In Sect. 4, we formally describe the algorithm from Theorem A and give a high level proof based on a number of lemmas which are proved in in subsequent chapters. Section 5 contains the proof of Theorem B. The rest of the paper contains several technical parts needed for the proof of Theorem A: in Sect. 6, we describe Berger’s effective Hurewicz inverse and analyze its running time (Theorem 1), in Sect. 7, we prove that the stages of the Whitehead tower have polynomial-time contractible loops (Lemma 4). Finally, in Sect. 8, we show how to reduce the case when the input is a simplicial complex $X^{sc}$ to the case of an associated simplicial set *X* and convert a map ${\varSigma }\rightarrow X$ into a map from a subdivision $Sd({\varSigma })$ into $X^{sc}$ (Lemma 6).

## Outline of the algorithm

In this section we present a high-level description of the main steps and ingredients involved in the algorithm from Theorem A.

### The algorithm in a nutshell

1. In the simplest case when the space *X* is $(d-1)$-connected (i.e., $\pi _i({X})=0$ for all $i\le d-1$), the classical Hurewicz Theorem (Hatcher 2001, Sect. 4.2) yields an isomorphism $\pi _d(X)\cong H_d(X)$ between the *d*th homotopy group and the *d*th homology group of *X*. Computing generators of the homology group is known to be a computationally easy task (it amounts to solving a linear system of equations over the integers). The key is then converting the homology generators into the corresponding homotopy generators, i.e., to compute an inverse of the Hurewicz isomorphism. This was described in the work of Berger (1991, 1995). We analyze the complexity of Berger’s algorithm in detail and show that it runs in exponential time in the size of *X* (assuming that the dimension *d* is fixed).
2. For the general case, we construct an auxiliary simplicial set $F_d$ together with a simplicial map $\psi _d: F_d\rightarrow {X}$ that has the following properties:
  $F_d$ is a simplicial set that is $d-1$ connected, and
  $\psi _d: F_d \rightarrow {X}$ induces an isomorphism $\psi _{d*} : \pi _d(F_d) \rightarrow \pi _d({X})$.
  Our construction of $F_d$ is based on computing stages of the Whitehead tower^5^ of *X* (Hatcher 2001, p. 356); this is similar to Real’s algorithm, which computes $\pi _d(X)$ as $H_d(F_d)$ as an abstract abelian group.
  The overall strategy is to use Berger’s algorithm on the space $F_d$ and compute generators of $\pi _d(F_d)$ as simplicial maps. Then we use the simplicial map $\psi _d$ to convert each generator of $\pi _d(F_d)$ into a map ${\varSigma }^d\rightarrow {X}$, and these maps generate $\pi _d({X})$. The main technical task for this step is to show that Berger’s algorithm can be applied to $F_d$. For this, we need to construct a polynomial algorithm for explicit contractions of loops in $F_d$ (this space is 1-connected but not 1-reduced in general).

### Our contributions

The main ingredients of the algorithm outlined above are the computability of stages of the Whitehead tower (Real 1996) as simplicial sets with polynomial-time homology and Berger’s algorithmization of the inverse Hurewicz isomorphism (Berger 1991, 1995).

The idea that these two tools can be combined to compute explicit representatives of $\pi _d(X)$ is rather natural and is also mentioned, for the special case of 1-reduced simplicial sets, in Romero and Sergeraert (2016, p. 3); however, there are a number of technical challenges to overcome in order to carry out this program. On a technical level, our main contributions are as follows:

- We give a complexity analysis of Berger’s algorithm to compute the inverse of the Hurewicz isomorphism (Theorem 1).
- We show that the homology generators of the Whitehead stage $F_d$ can be computed in polynomial time (Lemma 3).
- Berger’s algorithm requires an explicit algorithm for loop contraction—a certificate of 1-connectedness of the space $F_d$. While $F_d$ is not 1-reduced in general, we describe an explicit algorithm for contracting its loop and show that Berger’s algorithm can be applied.

We remark that the Whitehead tower stages are simplicial sets with infinitely many simplices, and we need the machinery of objects with polynomial-time homology to carry out the last two steps.

## Definitions and preliminaries

In this section, we give the necessary technical definitions that will be used throughout this paper. In the first part, we recall the standard definitions for simplicial sets and the toolbox of effective homology.

Afterwards, we present Kan’s definition of a loop space and further formalize our definition of (polynomial-time) loop contractions.

### Simplicial sets and polynomial-time effective homology

#### Simplicial sets and their computer representation

A simplicial set *X* is a graded set *X* indexed by the non-negative integers together with a collection of mappings $d_i :X_{{n}} \rightarrow X_{{n}-1}$ and $s_i :X_{n}\rightarrow X_{{n}+1}, \, 0\le i\le {n}$ called the *face* and *degeneracy* operators. They satisfy the following identities:

$$\begin{aligned} \begin{array}{ll} d_i d_j = d_{j-1}d_i &{}\quad \text {for } i<j, \\ d_i s_i = d_{i +1} s_i = {\mathrm{id}}&{} \quad \text {for } 0\le i< n, \\ d_i s_j = s_j d_{i-1} &{}\quad \text {for } i>j+1, \\ d_i s_j = s_{j-1} d_i &{}\quad \text {for }i<j,\\ s_i s_j = s_{j+1}s_i &{}\quad \text {for } i\le j.\\ \end{array} \end{aligned}$$

More details on simplicial sets and the motivation behind these formulas can be found in May (1992) and Goerss and Jardine (1999).

Simplicial maps between simplicial sets are maps of graded sets which commute with the face and degeneracy operators. The elements of $X_{n}$ are called ${n}$-*simplices*. We say that a simplex $x \in X_{n}$ is *(non-)degenerate* if it can(not) be expressed as $x = s_i y$ for some $y \in X_{{n}-1}$. If a simplicial set *X* is also a graded (Abelian) group and face and degeneracy operators are group homomorphisms, we say that *X* is a simplicial (Abelian) group.

A simplicial set is called *k*-reduced for $k\ge 0$ if it has a single *i*-simplex for each $i\le k$.

For a simplicial set *X*, we define the chain complex $C_* (X)$ to be a free Abelian group generated by the elements of $X_n$ with differential

$$\begin{aligned} \partial (c) = \sum _{i = 0} ^n (-1)^i d_i (c). \end{aligned}$$

A simplicial set is *locally effective* if its simplices have a specified finite encoding and algorithms are given that compute the face and degeneracy operators. A simplicial map *f* between locally effective simplicial sets *X* and *Y* is *locally effective* if an algorithm is given that for the encoding of any given $x\in X$ computes the encoding of $f(x)\in Y$.

We define a simplicial set to be *finite* if it has finitely many non-degenerate simplices. Such simplicial set can be algorithmically represented in the following way. The encoding of non-degenerate simplices can be given via a finite list and the encoding of a degenerate simplex $s_{i_k}\ldots s_{i_1} y$ for $i_1<i_2<\cdots <i_k$ and a non-degenerate *y* can be assumed to be a pair consisting of the sequence $(i_1,\ldots , i_k)$ and the encoding of *y*. The face operators are fully described by their action on non-degenerate simplices and can be given via finite tables. In this way, any simplicial set with finitely many non-degenerate simplices is naturally locally effective. Any choice of an implementation of the encoding and face operators is called a *representation* of the simplicial set. The *size of a representation* is the overall memory space one needs to store the data which represent the simplicial set.

#### Geometric realization

To each simplicial set *X* we assign a topological space |*X*| called its geometric realization. The construction is similar to that of simplicial complexes. Let ${\varDelta }_j$ be the geometric realization of a standard *j*-simplex for each $j\ge 0$. For each *k*, we define $D_i: {\varDelta }_{k-1}\hookrightarrow {\varDelta }_k$ to be the inclusion of a $(k-1)$-simplex into the *i*’th face of a *k*-simplex and $S_i: {\varDelta }_k\rightarrow {\varDelta }_{k-1}$ be the geometric realization of a simplicial map that sends the vertices $(0,1,\ldots ,k)$ of ${\varDelta }_k$ to the vertices $(0,1,\ldots ,i,i,i+1,\ldots ,k-1)$. The geometric realization |*X*| is then defined to be a disjoint union of all simplices *X* factored by the relation $\sim$

$$\begin{aligned} |X|:=\left( \bigsqcup _{n=0}^\infty X_n\times {\varDelta }_n\right) /\sim \end{aligned}$$

where $\sim$ is the equivalence relation generated by the relations $(x,D_i(p))\sim (d_i(x),p)$ for $x \in X_{n+1}$, $p \in {\varDelta }_n$ and the relations $(x, S_i(p))\sim (s_i(x), p)$ for $x \in X_{n-1}$, $p\in {\varDelta }_n$.

Similarly, a simplicial map between simplicial complexes naturally induces a continuous map between their geometric realizations.

#### Simplicial complexes and simplicial sets

In any simplicial complex $X^{sc}$, we can choose an ordering of vertices and define a simplicial sets $X^{ss}$ that consists of all non-decrasing sequences of points in $X^{sc}$: the dimension of $(V_0,\ldots , V_d)$ equals *d*. The face operator is $d_i$ omits the *i*’th coordinate and the degeneracy $s_j$ doubles the *j*’th coordinate. Moreover, choosing a maximal tree *T* in the 1-skeleton of *X* enables us to construct a simplicial set ${X}:=X^{ss}/T$ in which all vertices and edges in the tree, as well as their degeneracies, are considered to be a base-point (or its degeneracies). The geometric realizations of $X^{sc}$ and *X* are homotopy equivalent and *X* is 0-reduced, i.e. it has one vertex only.

#### Homotopy groups

Let $(X,x_0)$ be a pointed topological space. The *k*-th homotopy group $\pi _k(X,x_0)$ of $(X,x_0)$ is defined as the set of pointed homotopy^6^ classes of pointed continuous maps $({S}^k , *) \rightarrow (X,x_0)$, where $* \in {S}^k$ is a distinguished point. In particular, the 0-th homotopy group has one element for each path connected component of *X*. For $k=1$, $\pi _1(X,x_0)$ is the fundamental group of *X*, once we endow it with the group operation that concatenates loops starting and ending in $x_0$. The group operation on $\pi _k(X,x_0)$ for $k>1$ assigns to [*f*], [*g*] the homotopy class of the composition $S^k{\mathop {\rightarrow }\limits ^{\pi }} S^k\vee S^k {\mathop {\rightarrow }\limits ^{f\vee g}} X$ where $\pi$ factors an equatorial $(k-1)$-sphere containing $x_0$ into a point. Homotopy groups $\pi _k$ are commutative for $k>1$.

If the choice of base-points is understood from the context or unimportant, we will use the shorter notation $\pi _k(X)$. For a simplicial set *X*, we will use the notation $\pi _k(X)$ for the *k*’th homotopy group of its geometric realization |*X*|.

An important tool for computing homotopy groups is the *Hurewicz theorem*. It says that whenever *X* is $(d-1)$-connected, then there is an isomorphism $\pi _d(X)\rightarrow H_d(X)$. Moreover, if the element of $\pi _d(X)$ is represented by a simplicial map $f: {\varSigma }^d\rightarrow X$ and $\sum _j k_j \sigma _j$ represents a homology generator of $H_d({\varSigma }^d)$, then the Hurewicz isomorphism maps [*f*] to the homology class of the formal sum $\sum _{j} k_j f(\sigma _j)$ of *d*-simplices in *X*.

#### Effective homology

We call a chain complex $C_*$*locally effective* if the elements $c\in C_*$ have finite (agreed upon) encoding and there are algorithms computing the addition, zero, inverse and differential for the elements of $C_*$.

A locally effective chain complex $C_*$ is called *effective* if there is an algorithm that for given $n \in {\mathbb {N}}$ generates a finite basis $c_\alpha \in C_n$ and an algorithm that for every $c\in C_*$ outputs the unique decomposition of *c* into a linear combination of $c_\alpha$’s.

Let $C_*$ and $D_*$ be chain complexes. A *reduction* is a triple (*f*, *g*, *h*) of maps such that $f: C_*\rightarrow D_*$ and $g: D_*\rightarrow C_*$ are chain homomorphisms, $h: C_*\rightarrow C_*$ has degree 1, $fg=\mathrm {id}$ and $fg-\mathrm {id}=h\partial + \partial h$, and further $hh=hg=fh=0$.

A locally effective chain complex $C_*$ has *effective homology* ($C_*$ is a *chain complex with effective homology*) if there is a locally effective chain complex ${\tilde{C}}_*$, reductions  where $C_* ^\mathrm {ef}$ is an effective chain complex, and all the reduction maps are computable.

#### Eilenberg–MacLane spaces

Let $d\ge 1$ and $\pi$ be an Abelian group. An Eilenberg–MacLane space $K(\pi , d)$ is a topological space with the properties $\pi _d(K(\pi ,d))\simeq \pi$ and $\pi _j(K(\pi ,d))=0$ for $0<j\ne d$. It can be shown that such space $K(\pi ,d)$ exists and, under certain natural restrictions, has a unique homotopy type. If $\pi$ is finitely generated, then $K(\pi ,d)$ has a locally effective simplicial model (Krčál et al. 2013).

#### Globally polynomial-time homology and related notions

In many auxiliary steps of the algorithm, we will construct various spaces and maps. To analyze the overall time complexity, we need to parametrize all these objects by the very initial input, which is in our case an encoding of a finite 1-reduced simplicial set (or, in Theorem A.1, a more general space endowed with certain explicit certificate of 1-connectedness).

More generally, let ${\mathcal {I}}$ be a parameter set so that for each $I\in {\mathcal {I}}$ an integer $\mathrm {size}(I)$ is defined. We say that *F* is a parametrized simplicial set (group, chain group, ...) if for each $I\in {\mathcal {I}}$, a locally effective simplicial set (group, chain group, ...) *F*(*I*) is given. The simplicial set *F* is *locally polynomial-time* if there exists a locally effective model of *F*(*I*) such that for each $k\in {\mathbb {N}}$ and an encoding of a *k*-simplex $x\in F(I)$, the encoding of $d_i(x)$ and $s_j(x)$ can be computed in time polynomial in $\mathrm {size}(\text {enc}(x))+\mathrm {size}(I)$. The polynomial, however, may depend on *k*. A polynomial-time map between parametrized simplicial sets *F* and *G* is an algorithm that for each $k\in {\mathbb {N}}$, $I\in {\mathcal {I}}$ and an encoding of an *k*-simplex *x* in *F*(*I*) computes the encoding of *f*(*x*) in time polynomial in $\mathrm {size}(\text {enc}(x))+\mathrm {size}(I)$: again, the polynomial may depend on *k*.

Similarly, a locally polynomial-time (parametrized) chain complex is an assignment of a computer representation $C_*(I)$ of a chain complex with a distinguished basis in each gradation, such that all these basis elements have some agreed-upon encoding. A chain $\sum _j k_j \sigma _j$ is assumed to be represented as a list of pairs $(k_j, \text {enc}(\sigma _j))_j$ and has size $\sum _j (\mathrm {size}(k_j)+\mathrm {size}(\text {enc}(\sigma _j)))$, where we assume that the size of an integer $k_j$ is its bit-size. Further, an algorithm is given that computes the differential of a chain $z\in C_k(I)$ in time polynomial in $\mathrm {size}(z)+\mathrm {size}(I)$, the polynomial depending on *k*. The notion of a polynomial-time chain map is straight-forward.

A *globally polynomial-time chain complex* is a locally polynomial-time chain complex *EC* that in addition has all chain groups $EC(I)_k$ finitely generated and an additional algorithm is given that for each *k* computes the encoding of the generators of $EC(I)_k$ in time polynomial in $\mathrm {size}(I)$. Finally, we define a *simplicial set with globally polynomial-time homology* to be a locally polynomial-time parametrized simplicial set *F* together with reductions  where ${\tilde{C}},EC$ are locally polynomial-time chain complexes, *EC* is a globally polynomial-time chain complex and the reduction data are all polynomial-time maps, as usual the polynomials depending on the grading *k*.

The name “polynomial-time homology” is motivated by the following:

##### Lemma 1

Let *F* be a parametrized simplicial set with polynomial-time homology and $k\ge 0$ be fixed. Then all generators of $H_k(F(I))$ can be computed in time polynomial in $\mathrm {size}(I)$.

##### Proof

For the globally polynomial-time chain complex *EF* and each fixed *j*, we can compute the matrix of the differentials $d_j : EF(I)_j \rightarrow EF(I)_{j-1}$ with respect to the distinguished bases in time polynomial in $\mathrm {size}(I)$: we just evaluate $d_k$ on each element of the distinguished basis of $EF(I)_k$. Then the homology generators of $H_k(EC)$ can be computed using a Smith normal form algorithm applied to the matrices of $d_k$ and $d_{k+1}$, as is explained in standard textbooks (such as Munkres 1984). Polynomial-time algorithms for the Smith normal form are nontrivial but known (Kannan and Bachem 1981).

Let $x_1,\ldots ,x_m$ be the cycles generating $H_k(EF(I))$. We assume that reductionsare given and all the reduction maps are polynomial. Thus we can compute the chains

$$\begin{aligned} fg'(x_1),fg'(x_2),\ldots , fg'(x_m) \end{aligned}$$

in polynomial time and it is a matter of elementary computation to verify that they constitute a set of homology generators for $H_k(F(I))$. $\square$

### Loop spaces and polynomial-time loop contraction

#### Principal bundles and loop group complexes

In the text we will frequently deal with principal twisted Cartesian products: these are simplicial analogues of principal fiber bundles. The definitions in this section come from Kan’s article (Kan 1958b).

We first define the Cartesian product $X \times Y$ of simplicial sets *X*, *Y*: The set of *n*-simplices $(X \times Y)_n$ consists of tuples (*x*, *y*), where $x \in X_n, x\in Y_n$. The face and degeneracy operators on $X \times Y$ are given by $d_i (x,y) = (d_i x, d_i y)$, $s_i (x,y) = (s_i x, s_i y)$.

##### Definition 1

(*Principal Twisted Cartesian product*) Let *B* be a simplicial set with a basepoint $b_0\in B_0$ and *G* be a simplicial group. We call a graded map (of degree $-1$) $\tau : B_{n+1} \rightarrow G_{n}, n \ge 0$ a *twisting operator* if the following conditions are satisfied:

- $$d_{n}\tau (b)=\tau (d_{n+1}b)^{-1}\tau (d_n b)$$
- $$d_i\tau (b)=\tau (d_{i}b )$$ for $$0\le i < {n}$$
- $$s_i\tau (b)=\tau (s_{i} b)$$, $$i < {n}$$, and
- $\tau (s_{n}b)=1_{n}$ for all $b \in B_{n}$ where $1_{n}$ is the unit element of $G_{n}$.

Let *B*, *G*, $\tau$ be as above. We will define a *twisted Cartesian product*$B\times _\tau G$ to be a simplicial set *E* with $E_{n}=B_{n}\times G_{n}$, and the face and degeneracy operators are also as in the Cartesian product, i.e. $d_i(b,g) = (d_i b, d_i g)$ , with the sole exception of $d_{n}$, which is given by

$$\begin{aligned} d_{n}(b,g):=(d_{n}b, \tau (b) d_{n}(g)), \ \ \ \ (b,g)\in B_{n}\times G_{n}. \end{aligned}$$

It is not trivial to see why this should be the right way of representing fiber bundles simplicially, but for us, it is only important that it works, and we will have explicit formulas available for the twisting operator for all the specific applications.

We remark that in the literature one can find multiple definitions of twisted operator and twisted product (May 1992; Kan 1958b; Berger 1991) and that they, in essence differ from each other based on the decision whether the twisting “compresses” the first two or the last two face operators. Here, we follow the same notation as in Berger (1991).

#### Dwyer–Kan loop group construction

A simplicial set *X* can be viewed as a discrete description of a topological space |*X*|. It is natural to ask whether one can give a discrete description of a loop space of |*X*|. It turns out there are multiple models that can be used. Here, we describe the Dwyer–Kan’s *G*-construction (Kan 1958b) and later in Sect. 6, we present another model which is due to Berger (1991). Before the formal definition, we give some geometric intuition

For any $n \ge 0$ one can define a graph where $X_{n+1}$ is the set of edges and $X_0$ is the set of vertices with source and target operators $s,t:X_{n+1} \rightarrow X_0$, defined by $s(\sigma ) = (d_0)^{n+1} \sigma$ and $t (\sigma ) = d_{n+1} (d_0)^n \sigma$. Further a relation $1 = s_n \sigma$ is added.

In short, any simplex $\sigma \in X_{n+1}$ is an (*n*-dimensional) edge which goes from its second-to-last vertex to its last vertex and the simplex degenerate along this edge is considered a trivial path.

The Dwyer–Kan loop groupoid *GX* is defined as a free simplicial groupoid (e.g. paths) on the graph described above. In the case *X* is a 0-reduced simplicial set, the paths all begin and end in the only vertex, making them loops and the space *GX* can defined as follows:

##### Definition 2

Let *X* be a 0-reduced simplicial set. Then we define *GX* to be a (non-commutative) simplicial group such that

- $GX_n$ has a generator ${\overline{\sigma }}$ for each $(n+1)$-simplex $\sigma \in X$ and a relation $\overline{s_{n} y}=1$ for each simplex in the image of the last degeneracy $s_{n}$.
- The face operators are given by $d_i {\overline{\sigma }}:=\overline{d_i \sigma }$ for $i<n$ and $d_n {\overline{\sigma }}:=(\overline{d_{n+1}\sigma })^{-1} \overline{d_n \sigma }$
- The degeneracy operators are $s_i {\overline{\sigma }}:=\overline{s_i \sigma }$.

We use the multiplicative notation, with 1 being the neutral element. For the proof that *GX* is indeed a discrete simplicial analog of the loop space of *X*, see Kan (1958b) and May (1992).

For algorithmic puroposes, we assume that an elements $\prod _{j} {\overline{\sigma }}_j^{k_j}$ of *GX* is represented as a list of pairs $(\sigma _j, k_j)$ and has size $\sum _j \mathrm {size}(\sigma _j)+\mathrm {size}(k_j)$.

##### Definition 3

Let *X* be a 0-reduced simplicial set. We say that a map $c_0: GX_0\rightarrow GX_1$ is a *contraction* of loops in *X* if $d_0 c_0(x)=x$ and $d_1 c_0(x)=1$ for each $x\in GX_0$.

In case where *X* has finitely many nondegenerate 1-simplices, we define the size $\mathrm {size}(c_0)$ to be the sum

$$\begin{aligned} \sum _{\gamma \in X_1} \mathrm {size}(c_0(\gamma )). \end{aligned}$$

#### Loop contraction for simplicial complexes

Let $X^{sc}$ be a simplicial complex. Let *T* be a spanning tree in the 1-skeleton of $X^{sc}$ and *R* a chosen vertex. For each oriented edge $e=(v_1 v_2)$ we define a formal inverse to be $e^{-1}:=(v_2 v_1)$ and we also consider degenerate edges (*v*, *v*). A *loop* is defined as a sequence $e_1,\ldots , e_k$ of oriented edges in $X^{sc}$ such that

- The end vertex of $e_i$ equals the initial vertex of $e_{i+1}$, and
- The initial vertex of $e_1$ and the end vertex of $e_k$ equal *R*.

Every edge *e* that is not contained in *T* gives rise to a unique loop $l_e$. Further, every loop in $X^{sc}$ is either a concatenation of such $l_e$’s, or can be derived from such concatenation by inserting and deleting consecutive pairs $(e,e^{-1})$ and degenerate edges. Before we formally define our combinatorial version of loop contraction, we need the following definition.

##### Definition 4

Let *S* be a set, $U\subseteq S$, *F*(*S*) and *F*(*U*) be free groups generated by *S*, *U*, respectively.^7^ Let $h_U: F(S)\rightarrow F(S)$ be a homomorphism that sends each $u\in U$ to 1 and each $s\in S{\setminus } U$ to itself. We say that an element *x* of *F*(*S*) equals *y* modulo *U* if $h_U(x)=y$.

An example of an element that is trivial modulo *U* is the word $s \, u \, s^{-1}$, where $s\in S$ and $u\in U$.

##### Definition 5

Let *S* be the set of all oriented edges and oriented degenerate edges in $X^{sc}$ and assume that a spanning tree *T* is chosen. Let *U* be the set of all oriented edges in *T*, including all degenerate edges. A *contraction of an edge* $\alpha$ is a sequence of vertices $A_0,A_1,\ldots ,A_s$ and $B_1,\ldots , B_{s}$ such that

- for each *i*, $\{A_i,A_{i+1}, B_{i+1}\}$ is a simplex of $X^{sc}$, and
- the element of *F*(*S*)
  1 $$\begin{aligned} (A_0 B_1) (B_1 A_1) (A_1 B_2) (B_2 A_2) \ldots (B_s A_s) (A_s A_{s-1}) (A_{s-1} A_{s-2}) \ldots (A_1 A_0) \end{aligned}$$
  equals $$\alpha $$ modulo *U*.
  A *loop contraction in a simplicial complex* is the choice of a contraction of $\alpha$ for each edge $\alpha \in X^{sc}{\setminus } T$.

The size of the contraction of $\alpha$ is defined to be the number of vertices in (1) and the size $\mathrm {size}(c)$ of the loop contraction on $X^{sc}$ is the sum of the sizes over all $\alpha \in X^{sc}{\setminus } T$.

**Fig. 1:**
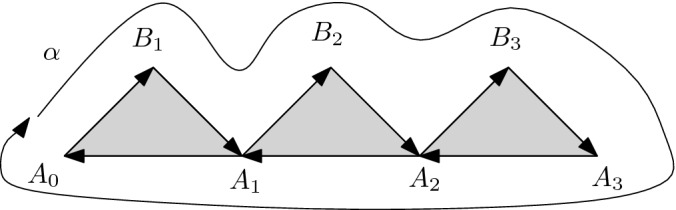
The loop ranging over the boundary of this geometric shape equals $\alpha$, after ignoring edges in the maximal tree and canceling pairs $(e,e^{-1})$. The interior of the triangles gives rise to a contraction

The geometry behind this definition is displayed in Fig. 1. The sequence of $A_i$’s and $B_j$’s gives rise to a map from the sequence of (full) triangles into $X^{sc}$. The big loop around the boundary is combinatorially described by (1). We can continuously contract all of its parts that are in the tree *T* to a chosen basepoint, as the tree is contractible. Further, we can continuously contract all pairs of edges $(e,e^{-1})$ and what remains is the original edge $\alpha$: with all the tree contracted to a point, it will be transformed into a loop that geometrically corresponds to $l_\alpha$. The interior of the full triangles then constitutes its “filler”, hence a certificate of the contractibility of $l_\alpha$.

A loop contraction in the sense of Definition 1 exists iff the space $X^{sc}$ is simply connected. One could choose different notions of loop contraction. For instance, we could provide, for each $\alpha$, a simplicial map from a triangulated 2-disc into $X^{sc}$ such that the oriented boundary of the disc would be mapped exactly to $l_\alpha$. The description from Definition 5 could easily be converted into such map. We chose the current definition because of its canonical and algebraic nature. The connection between Definitions 3 and 5 is the content of the following lemma.

##### Lemma 2

Let $X^{sc}$ be a 1-connected simplicial complex with a chosen orientation of all simplices, $X^{ss}$ the induced simplicial set, *T* a maximal tree in $X^{sc}$, and ${X}:=X^{ss}/T$ the corresponding 0-reduced simplicial set. Assume that a loop contraction in the simplicial complex $X^{sc}$ is given, such as described in Definition 5. Then we can algorithmically compute $c_0(\alpha )\in G{X}_1$ such that $d_0 c_0(\alpha )=\alpha$ and $d_1 c_0(\alpha )=1$, for every generator $\alpha$ of $G{X}_0$. Moreover, the computation of $c_0(\alpha )$ is linear in the size of $X^{sc}$ and the size of the simplicial complex contraction data.

##### Proof

For each *i*, the triangle $\{A_i,A_{i+1},B_{i+1}\}$ from Definition 5 is in the simplicial complex $X^{sc}$. There is a unique oriented 2-simplex in $X^{ss}$ of the form $(V_0,V_1,V_2)$ (possibly degenerate) such that $\{V_0,V_1,V_2\}=\{A_i,A_{i+1},B_{i+1}\}$. Let us denote such oriented simplex by $\sigma _i$, and its image in $G{X}_1$ by ${\overline{\sigma }}_i$. We will define an element $g_i\in G{X}_1$ such that it satisfies

2 $$\begin{aligned} d_0 g_i\simeq \overline{(A_i, A_{i+1})} \quad \text { and }\quad d_1 g_i\simeq \overline{(A_i, B_{i+1})}\,\, \overline{(B_{i+1},A_{i+1})} \end{aligned}$$

where $\simeq$ is an equivalence relation that identifies any element $\overline{(U,V)}\in G{X}_1$ with $\overline{(V,U)}^{-1}$ (note that only one of the symbols (*U*, *V*) and (*V*, *U*) is well defined in $X^{ss}$, resp. *X*.) Explicitly, we can define $g_i$ with these properties as follows:

- If $$\sigma _i=(B_{i+1}, A_i, A_{i+1})$$, then $$g_i:={\overline{\sigma }}_i$$,
- If $$\sigma _i=(A_i, A_{i+1}, B_{i+1})$$, then $$g_i:=s_0\overline{(d_2 {\sigma _i})} \, {\overline{\sigma }}_i \,s_0 d_0({\overline{\sigma }}_i)^{-1}$$
- If $$\sigma _i=(A_{i+1}, B_{i+1}, A_{i})$$, then $$g_i= s_0 d_0\overline{\sigma _i}^{-1} \,\overline{\sigma _i} \, s_0 (\overline{d_1 {\sigma }_i})^{-1}$$
- If $$\sigma _i=(B_{i+1}, A_{i+1}, A_i)$$, then $$g_i:=\overline{\sigma _i}^{-1}$$
- If $$\sigma _i=(A_{i+1}, A_i, B_{i+1})$$, then $$g_i:= s_0 d_0\overline{\sigma _i} \,\, \overline{\sigma _i}^{-1}\, s_0 (\overline{d_2 \sigma _i})^{-1}$$
- If $$\sigma _i=(A_{i}, B_{i+1}, A_{i+1})$$, then $$g_i:=s_0(\overline{d_1 \sigma _i})\,\overline{\sigma _i}^{-1} s_0 d_0\overline{\sigma _i}$$.

Let $g:=g_0\ldots , g_s$. The assumption (1) together with Eq. (2) immediately implies that $d_1 g (d_0 g)^{-1}={\overline{\alpha }}$. Thus we define $c_0({\overline{\alpha }}):=s_0 d_1(g)\,g^{-1}$. Algorithmically, to construct *g* amounts to going over all the triples $(A_{i}, A_{i+1},B_{i+1})$ from a given sequence of $A_i's$ and $B_j$’s, checking the orientation and computing $g_i$ for every *i*. $\square$

#### Polynomial-time loop contraction

Let *F* be a parametrized simplicial set such that each *F*(*I*) is 0-reduced. Using constructions analogous to those defined above, *GF* is a parametrized locally-polynomial simplicial group whereas we assume a simple encoding of elements of $GF_i$ as follows. If $x=\prod _j \overline{\sigma _j}^{k_j}\in GF(I)_k$ where $\sigma _j$ are $(k+1)$-simplices in *F*(*I*), not in the image of $s_k$, then we assume that *x* is stored in the memory as a list of pairs $(k_j,\text {enc}(\sigma _j))$ and has size $\sum _j (\mathrm {size}(k_j)+\mathrm {size}(\sigma _j))$ where some $\sigma _i$ may be equal to $\sigma _j$ for $i\ne j$. Face and degeneracy operators are defined in Definition (2) and it is easy to see that for any locally polynomial-time simplicial set *F*, *GF* is a locally polynomial-time simplicial group.

##### Definition 6

Let *F* be a locally polynomial simplicial set. We say that *F* has *polynomially contractible loops* if there exists an algorithm that for a 0-simplex $x\in GF(I)$ computes a 1-simplex $c_0(x)\in GF(I)$ such that $d_0 x=x$, $d_1 x=1 \in GF(I)_0$, and the running-time is polynomial in $\mathrm {size}(x)+\mathrm {size}(I)$.

## Proof of Theorem 1

We will prove a stronger statement of Theorem A formulated as follows.

### Theorem A.1

There exists an algorithm that, given $d\ge 2$ and a finite 0-reduced simplicial set *X* (alternatively, a finite simplicial complex) with an explicit loop contraction $c_0$ (such as in Definitions 3 or 5) computes the generators $g_1,\ldots , g_k$ of $\pi _d(X)$ as simplicial maps ${\varSigma }_j^d\rightarrow X$, for suitable triangulations ${\varSigma }_j^d$ of $S^d$, $j=1,\ldots ,k$.

For fixed *d*, the time complexity is exponential in the *size* of *X* and the size of the loop contraction $c_0$; more precisely, it is $O(2^{P(\mathrm {size}(X)+\mathrm {size}(c_0))})$ where $P=P_d$ is a polynomial depending only on *d*.

This immediately implies Theorem A, as for a 1-reduced simplicial set, the contraction $c_0$ is trivial, given by $c_0(1)=1$.

The proof of Theorem A.1 is based on a combination of four statements presented here as Lemma 3, Theorem 1, Lemma 4 and Lemma 6. Each of them is relatively independent and their proofs are delegated to further sections.

First we present an algorithm that, given a 1-connected finite simplicial set *X* and a positive integer *d*, outputs a simplicial set $F_d$ and a simplicial map $\psi _d$ such that

- the simplicial set $F_d$ is $d-1$ connected, it has polynomial-time effective homology and polynomially contractible loops.
- the simplicial map $\psi _d: F_d \rightarrow X$ is polynomial-time and induces an isomorphism $\psi _{d*} : \pi _d(F_d) \rightarrow \pi _d(X)$.

### Whitehead tower

We construct simplicial sets $F_d$ as stages of a so-called *Whitehead tower* for the simplicial set *X*. It is a sequence of simplicial sets and mapswhere $f_i$ induces an isomorphism $\pi _j(F_{i+1}) \rightarrow \pi _j(F_{i})$ for $j>i$ and $\pi _j(F_i) = 0$ for $j< i$. We define $\psi _d = f_d f_{d-1} \ldots f_3$. One can see that $F_d, \psi _d$ satisfy the desired properties.

#### Lemma 3

  Let $d\ge 2$ be a fixed integer. Then there exists a polynomial-time algorithm that, for a given 1-connected finite simplicial set *X*, constructs the stages $F_2, \ldots , F_d$ of the Whitehead tower of *X*.

The simplicial sets $F_k(X)$, parametrized by 1-connected finite simplicial sets *X*, have polynomial-time homology and the maps $f_k$ are polynomial-time simplicial maps.

#### Proof

The proof is by induction. The basic step is trivial as $F_2 = X$. We describe how to obtain $F_{k+1}, f_{k+1}$ assuming that we have computed $F_{k}$, $2\le k <d$.

1. We compute simplicial map $\varphi _{k}: F_{k} \rightarrow K(\pi _{k}(X), k) = K(\pi _k(F_k), k)$ that induces an isomorphism $\varphi _{k*}: \pi _{k}(F_{k}) \rightarrow \pi _{k}(K(\pi _{k}(X), k))\cong \pi _{k}(X)$. This is done using the algorithm in Čadek et al. (2014b), as $K(\pi _{k}(X), k)$ is the first nontrivial stage of the Postnikov tower for the simplicial set $F_{k}$.
  For the simplicial set $K(\pi _{k}(X), k)$ and for such simplicial sets there is a classical principal bundle (twisted Cartesian product) (see May 1992):
2. We construct $F_{k+1}$ and $f_{k+1}$ as a pullback of the twisted Cartesian product:

It can be shown that the pullback, i.e. simplicial subset of pairs $(x,y) \in F_k \times E(\pi _{k}(X),k-1)$ such that $\delta (y) = \varphi _k(x)$, can be identified with the twisted product as above (May 1992), where the twisting operator $\tau '$ is defined as $\tau \varphi _k$.

To show correctness of the algorithm, we assume inductively, that $F_{k}$ has polynomial-time effective homology. According to Čadek et al. (2014b, Section 3.8), the simplicial sets $K(\pi _{k}(X), {k-1})$, $E(\pi _{k}(X), k-1)$, $K(\pi _{k}(X), k)$ have polynomial-time effective homology and maps $\varphi _k, \delta$ are polynomial-time. Further, they are all obtained by an algorithm that runs in polynomial time.

As $F_{k+1}$ is constructed as a twisted product of $F_k$ with $K(\pi _{k}(X), k)$, Corollary 3.18 of Čadek et al. (2014b) implies that $F_{k+1}$ has polynomial-time effective homology and $f_{k+1}$ is a polynomial-time map.^8^

The sequence of simplicial sets  induces the long exact sequence of homotopy groupsThe reason why this is the case follows from a rather technical argument that identifies the simplicial set $F_{k +1}$ with a so called *homotopy fiber* of the map $\varphi _{k}: F_{k} \rightarrow K (\pi _{k}(X), k)$. In more detail, the category of simplicial sets is right proper (Goerss and Jardine 1999, II.8.67) and map $\delta$ is a so-called Kan fibration (May 1992, §23). This makes the pullback $F_{k +1 }$ coincide with so-called homotopy pullback. Further, the simplicial set $E(\pi _{k}(X),k-1)$ is contractible, hence the homotopy pullback is a homotopy fiber. The induced exact sequence is due to Quillen (1967, chapter I.3).

The inductive assumption, together with the fact that $\varphi _k$ induces an isomorphism $\varphi _{k*}: \pi _{k}(F_{k}) \rightarrow \pi _{k}(K(\pi _{k}(X),k))$ imply that $f_k$ induces an isomorphism $\pi _j(F_{k +1}) \rightarrow \pi _j(F_{k})$ for $j>k$ and $\pi _j(F_{k +1}) = 0$ for $j\le k$. $\square$

The lemma implies that the simplicial sets $F_k$ have polynomial-time effective homology and maps $\psi _k = f_k f_{k-1} \ldots f_3$ are polynomial-time as they are defined as a composition of polynomial-time maps $f_i$.

The following theorem is a key ingredient of our algorithm.

#### Theorem 1

(Effective Hurewicz Inverse) Let $d>1$ be fixed and *F* be an $(d-1)$-connected 0-reduced simplicial set parametrized by a set ${\mathcal {I}}$, with polynomial-time homology and polynomially contractible loops.

Then there exists an algorithm that, for a given *d*-cycle $z\in Z_{d}(F(I))$, outputs a simplicial model ${\varSigma }^{d}$ of the *d*-sphere and a simplicial map ${\varSigma }^{d}\rightarrow F(I)$ whose homotopy class is the Hurewicz inverse of $[z]\in H_{d}(F(I))$.

Moreover, the time complexity is bounded by an exponential of a polynomial function in $\mathrm {size}(I)+\mathrm {size}(z)$.

The construction of an effective Hurewicz inverse is the main result of Berger (1991) and further details are provided in Sect. 6. It exploits a combinatorial version of Hurewicz theorem given by Kan (1958a) where $\pi _d(F)$ is described in terms of $\pi _{d-1}({\widetilde{GF}})$ where ${\widetilde{GF}}$ is a non-commutative simplicial group that models the loop space of *F*. Kan showed that the Hurewicz isomorphism can be identified with a map $H_{d-1}({\widetilde{GF}})\rightarrow H_{d-1}({\widetilde{AF}})$ induced by Abelianization. Berger then describes the inverse of the Hurewicz homomorphism as a composition of the maps 1, 2, 3 in the diagramArrow 1 is induced by a chain homotopy equivalence and arrow 3 by Berger’s explicit geometric model of the loop space. To algorithmize arrow 2, we need an algebraic machinery that includes an explicit contraction of *k*-loops in ${\widetilde{GF}}$ for all $k<d-1$. Those are based partially on linear computations in the Abelian group ${\widetilde{AF}}$ and partially on explicit inductive formulas dealing with commutators. The lowest-dimensional contraction operation, however, cannot be algorithmized, without some external input. The possibility of providing it is the content of the following claim:

#### Lemma 4

Let $d\ge 2$ be a fixed integer and ${\mathcal {I}}$ be the set of all 1-connected 0-reduced finite simplicial sets with an explicit loop contraction $c_0$. Then the simplicial set $F_d$ from Lemma 3, parametrized by ${\mathcal {I}}$ has polynomial-time contractible loops (see Definition 6).

The proof is constructive, based on explicit formulas in our model of $F_d$. The details are in Sect. 7.

We remark that the output of the algorithm in Lemma 4 i.e. the loop contraction of $F_d$ is polynomial time with respect to the input—a 0-reduced and 1-connected simplicial set with a specific loop contraction $c_0$ on this simplicial set.

The core of the algorithm we will describe works with simplicial sets and simplicial maps between them. If our input is a simplicial complex, we need tools to convert them into maps between simplicial complexes. The next two lemmas address this.

#### Lemma 5

Let *Y* be a finite simplicial set. Then there exists a polynomial-time algorithm that computes a simplicial complex $Y^{sc}$ with a given orientation of each simplex, and a map $\gamma : Y^{sc}\rightarrow Y$ (still understood to be a map between simplicial sets) such that the geometric realization of $\gamma$ is homotopic to a homeomorphism.

This construction is originally due to Barratt (1956), and described in detail in Čadek et al. (2013b, Appendix B).^9^ Explicitly, the simplicial complex $Y^{sc}$ is defined to be $Y^{sc}:=B_*(Sd(Y))$, where *Sd* is the barycentric subdivision functor and $B_*$ a functor introduced in Jardine (2004): $Y^{sc}$ can be constructed recursively by adding a vertex $v_\sigma$ for each nondegenerate simplex $\sigma \in Sd(Y)$ and replacing $\sigma$ by the cone with apex $v_\sigma$ over $B_*(\partial \sigma )$. The subdivision *Sd*(*Y*) is a regular simplicial set and $B_*(Sd(Y))$ coincides with the flag simplicial complex of the poset of nondegenerate simplices of *Sd*(*Y*). It follows that the geometric realizations $|Y^{sc}|$ is homeomorphic^10^ to |*Y*|. Simplices of $Y^{sc}$ are naturally oriented and the explicit description of $\gamma$ is given in Čadek et al. (2013b, p. 61) and the references therein.

In our main algorithm, $Y={\varSigma }^d$ will be a triangulation of the *d*-sphere and *X* a simplicial set derived from a simplicial complex $X^{sc}$ by contracting its spanning tree into a point. The following lemma shows that we can convert a map ${\varSigma }^{sc}\rightarrow X$ into a map $({\varSigma }^{sc})'\rightarrow X^{sc}$ between simplicial complexes.

#### Lemma 6

Let $d>0$ be fixed. Assume that $X^{sc}$ is a given simplicial complex with a chosen ordering of vertices and a maximal spanning tree *T*; we denote the underlying simplicial set by $X^{ss}$. Let $p: X^{ss} \rightarrow X:=X^{ss}/T$ be the projection to the associated 0-reduced simplicial set. Let ${\varSigma }$ be a given *d*-dimensional simplicial complex with a chosen orientation of each simplex, ${\varSigma }^{ss}$ the induced simplicial set, and $f:{\varSigma }^{ss}\rightarrow {X}$ a simplicial map.

Then there exists a subdivision $\mathrm {Sd}({\varSigma })$ and a simplicial map $f':\mathrm {Sd}({\varSigma })\rightarrow X^{sc}$ between *simplicial complexes*^11^ such that

$$\begin{aligned} |{\varSigma }|=|\mathrm {Sd}({\varSigma })| {\mathop {\rightarrow }\limits ^{|f'|}} |X^{sc}| {\mathop {\rightarrow }\limits ^{|p|}} |X| \end{aligned}$$

is homotopic to $|{\varSigma }^{ss}|{\mathop {\rightarrow }\limits ^{|f|}} |{X}|$. Moreover, $f'$ can be computed in polynomial time, assuming an encoding of the input $f,{\varSigma },X^{sc}$, *X* and *T*.

Thus if ${\varSigma }$ is a sphere and *f* corresponds to a homotopy generator, $f'$ is the corresponding homotopy generator represented as a simplicial map between simplicial complexes. We remark that the algorithm we describe works even if *d* is a part of the input, but the time complexity would be exponential in general, as the number of vertices in our subdivision $\mathrm {Sd}({\varSigma })$ would grow exponentially with *d*.

The proof of Lemma 6 is given in Sect. 8.

#### Proof of Theorem A.1

First assume that a finite simplicial complex $X^{sc}$ is given together with a loop contraction. Then the algorithm goes as follows.

1. We choose an ordering of vertices and convert $X^{sc}$ into a simplicial set. Choosing a spanning tree and contracting it to a point creates a 0-reduced simplicial set *X* homotopy equivalent to $X^{sc}$. By Lemma 2, we can convert the input data into a list $c_0(\alpha )$ for all generators $\alpha$ of $GX_0$ in polynomial time.
2. We construct the simplicial set $F_d$ from Lemma 3 as simplicial set with polynomial-time effective homology. Hence by Lemma 1 we can compute the generators of $H_d(F_d)$ in time polynomial in $\mathrm {size}(X)$. Due to Lemma 4 and Theorem 1, we can convert these homology generators to homotopy generators ${\varSigma }_j^d \rightarrow F_d$ in time exponential in $P(\mathrm {size}(X)+\mathrm {size}(c_0))$ where *P* is a polynomial.
3. We compose the representatives of $\pi _d(F_d)$ with $\psi _d$ to obtain representatives ${\varSigma }_j^d\rightarrow X$ of the generators of $\pi _d(X)$, another polynomial-time operation. This way, we compute explicit homotopy generators as maps into the simplicial set *X*.
4. We use Lemma 5 to compute simplicial complexes ${\varSigma }_j^{sc}$ and maps ${\varSigma }_j^{sc}\rightarrow {\varSigma }^d$ homotopic to homeomorphisms. The compositions ${\varSigma }_j^{sc}\rightarrow {\varSigma }_j^d\rightarrow X$ still represent a set of homotopy generators. Finally, by Lemma 6, we can compute, for each *j*, a subdivision of the sphere ${\varSigma }_j^{sc}$ and a simplicial map from this subdivision into the simplicial *complex*$X^{sc}$, in time polynomial in the size of the representatives ${\varSigma }_j^{sc}\rightarrow X$.

In case when the input is a 0-reduced simplicial set *X* with a loop contraction $c_0$, only steps 2 and 3 are performed. In either case, the overall exponential complexity bound comes from Berger’s Effective Hurewicz inverse theorem. $\square$

## Proof of Theorem B

Similarly as in the proof of Theorem A, we prove a slightly more general version of Theorem B that also includes finite simplicial complexes.

### Theorem B.1

Let $d\ge 2$ be fixed. Then

1. there is an infinite family of *d*-dimensional 1-connected finite simplicial complexes *X* such that for any simplicial map ${\varSigma }\rightarrow X$ representing a generator of $\pi _d(X)$, the triangulation ${\varSigma }$ of $S^d$ on which *f* is defined has size at least $2^{{\varOmega }(\mathrm {size}(X))}$.
2. there is an infinite family of *d*-dimensional $(d-1)$-connected and $(d-2)$-reduced simplicial sets *X* such that for any simplicial map ${\varSigma }\rightarrow X$ representing a generator of $\pi _d(X)$, the triangulation ${\varSigma }$ of $S^d$ on which *f* is defined has size at least $2^{{\varOmega }(\mathrm {size}(X))}$.

Consequently, any algorithm for computing simplicial representatives of the generators of $\pi _d(X)$ has time complexity at least $2^{{\varOmega }(\mathrm {size}(X))}$.

The second item immediately implies Theorem B.

In the first item, we don’t assume any certificate for 1-connectedness. However, we suspect that any algorithm that computes representatives of $\pi _d(X)$ for simplicial complexes *Xmust* necessarily use some explicit certificate of simple connectivity, but so far we have not been able to verify this.

### Lemma 7

Let $$d\ge 2$$.

1. There exists a sequence $\{X_k\}_{k \ge 1}$ of *d*-dimensional $(d-1)$-connected simplicial complexes, such that $H_{d}(X_k)\simeq {\mathbb {Z}}$ for all *k* and for any choice of a cycle $z_k\in Z_{d}(X_k)$ generating the homology group, the largest coefficient in $z_k$ grows exponentially in $\mathrm {size}(X_k)$.
2. There exists a sequence $\{X_k\}_{k \ge 1}$ of *d*-dimensional $(d-1)$-connected and $(d-2)$-reduced simplicial sets, such that $H_{d}(X_k)\simeq {\mathbb {Z}}$ for all *k* and for any choice of cycles $z_k\in Z_{d}(X_k)$ generating the homology, the largest coefficient in $z_k$ grows exponentially^12^ in $\mathrm {size}(X_k)$.

### Proof of Theorem 2 based on Lemma 7

Let $\{X_k\}_{k \ge 1}$ be the sequence of simplicial sets or simplicial complexes from Lemma 7. Since they are $(d-1)$-connected, by the theorem of Hurewicz, $\pi _{d}(X_k)\simeq H_{d}(X_k)\simeq {\mathbb {Z}}$. For each *k*, let ${\varSigma }_k$ be a simplicial set or simplicial complex with $|{\varSigma }_k|=S^{d}$, and $f_k: {\varSigma }_k\rightarrow X_k$ a simplicial map representing a generator of $\pi _{d}(X_k)$. The generator of $H_d({\varSigma }_d)$ contains each non-degenerate *d*-simplex with a coefficient $\pm 1$ (this follows from the fact that ${\varSigma }_k$ is a triangulation of the *d*-sphere and the *d*-homology of the *d*-sphere is generated by its fundamental class). The Hurewicz isomorphism $\pi _{d}(X_k)\rightarrow H_{d}(X_k)$ maps such a representative to the formal sum of simplices

$$\begin{aligned} f_k \mapsto \sum _{\sigma \,\text { is a } d-\text {simplex in } ({\varSigma }_k)} \pm f_k(\sigma ) \in C_{d}(X_k) \, , \end{aligned}$$

which represents a generator of $H_{d}(X_k)$. It follows from Lemma 7 that the number of *d*-simplices in ${\varSigma }_k$ grows exponentially in $\mathrm {size}(X_k)$. Moreover, the complexity of any algorithm that computes $f_k: {\varSigma }_k\rightarrow X_k$ is at least the size of ${\varSigma }_k$, which completes the proof. $\square$

It remains to define the sequence from Lemma 7:

### Proof of Lemma 7

1. We begin by constructing for every $d\ge 2$, a sequence of $\{X_k\}_{k \ge 1}$ of $(d-1)$-connected simplicial complexes, such that $H_{d}(X_k)\simeq {\mathbb {Z}}$ for all *k*, and for any choice of a cycle $z_k\in Z_{d}(X_k)$ generating the homology group, the largest coefficient in $z_k$ grows exponentially in $\mathrm {size}(X_k)$.

**Fig. 2:**
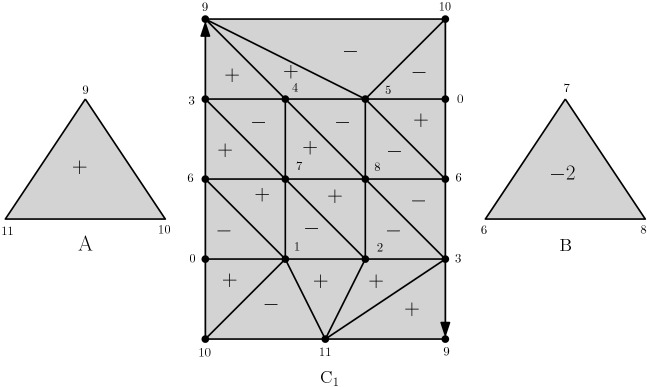
The Möbius band is the mapping cylinder of a degree 2 map $S^1 \rightarrow S^1$. The triangulation has four layers because starting from the boundary, which is a triangle, we first need to pass to a hexagon in order to cover the middle triangle twice, obtaining the desired degree 2 map. Connecting *k* copies of the Möbius band creates a mapping cylinder of a degree $2^k$ map, using only linearly (in *k*) many simplices. Gluing the full triangles *A* and *B* to the ends of this mapping cylinder finishes the construction of $X_k$. The red coefficients exhibit a generator $\xi$ of $H_2(X_1) = Z_2 (X_1) \simeq {\mathbb {Z}}$ given as a formal sum of 2-simplices

We start with $d=2$. The idea is to glue $X_k$ out of *k* copies of a triangulated mapping cylinders of a degree 2 map ${S}^1 \rightarrow {S}^1$, i.e. *k* Möbius bands, and then fill in the two open ends with one triangle each (*A* and *B* in Fig. 2). The case $k=1$ is shown in Fig. 2. For $k \ge 2$, we take *k* copies of the triangulated Möbius band and identify the middle circle of each one to the boundary of the next one.

We observe that, up to homotopy equivalence, $X_k$ consists of a 2-disc with another 2-disc which is attached to it via the boundary map $S^1\rightarrow S^1$ of degree $2^k$. Therefore, $X_k$ is simply connected and has $H_2(X_k) \simeq {\mathbb {Z}}$ and any homology generator will contain the 2-simplex *A* with coefficient $\pm 1$ and *B* with coefficient $\pm 2^k$.

Similarly for $d>2$, the simplicial complex $X_k$ is obtained by glueing *k* copies of a triangulated mapping cylinder of a degree 2 map $S^{d-1} \rightarrow S^{d-1}$, and the two open ends are filled in with two triangulated *d*-balls.

2. For every $k \ge 1$ we define the simplicial sets $X_k$ to have one vertex $*$, no non-degenerate simplices up to dimension $d-2$, *k* non-degenerate $(d-1)$-simplices $\sigma _1,\ldots , \sigma _k$ that are all spherical (that is, for all *i*, *j*, $d_i \sigma _j=*$ is the degeneracy of the only vertex of $X_k$), and $k+1 \,$*d*-simplices $A,C_1,C_2,\ldots , C_{k-1},B$ such that

- $$d_0 A=\sigma _1$$, $$d_j A=*$$ for $$j>0$$,
- $d_0 C_i=\sigma _i$, $d_1 C_i=\sigma _{i+1}$, $d_2 C_i=\sigma _i$ and $d_j C_i=*$ for $j>2$, and
- $$d_{0} B=\sigma _k$$, $$d_j B=*$$ for $$j>0$$.

$X_k$ does not have any non-degenerate simplices of dimension larger than *d*. The relations of a simplicial set are satisfied, because $d_i d_j$ is trivial in all cases.

The boundary operator in the associated normalised chain complex $C_*(X_i)$ acts on basis elements as

- $$\partial A=\sigma _1$$
- $$\partial C_i=2\sigma _i-\sigma _{i+1}$$, and
- $$\partial B=\sigma _k$$.

To see that $X_k$ is $(d-1)$-connected for $d>2$, it is enough to prove that $H_{d-1}(X_k)$ is trivial (by 1-reduceness and Hurewicz theorem). This is true, because $\sigma _1$ is the boundary of *A* and for $i>1$, $\sigma _i$ is the boundary of the chain

$$\begin{aligned} 2^{i-1} A - 2^{i-2} C_1 - \cdots - 2 C_{i-2}-C_{i-1}. \end{aligned}$$

In the case $d=2$, $X_k$ is not 1-reduced, but we can show 1-connectedness similarly as in the proof of the first part: up to homotopy, $X_k$ consists of two discs with boundaries together via a map of degree $2^{k-1}$.

There are no non-degenerate $(d+1)$-simplices, so $H_{d}(X_k)\simeq Z_{d}(X_k)$ and a simple computation shows that every cycle is a multiple of

$$\begin{aligned} 2^{k-1} A - 2^{k-2} C_1 - 2^{k-3} C_2 -\cdots - C_{k-1} - B. \end{aligned}$$

The computer representation of $X_k$ has size that grows linearly with *k*, but the coefficients of homology generators grow exponentially with *k*, so they grow exponentially with $\mathrm {size}(X_k)$. $\square$

### Discussion on optimality

If $d=2$ and *X* is a 1-reduced simplicial set, then generators of $H_2(X)$ can be computed via the Smith normal form of the differential $\partial _3: C_3(X)\rightarrow C_2(X)$. Using canonical bases, the matrix of $\partial _3=d_0-d_1+d_2-d_3$ satisfies that the sum of absolute values over each column is at most 4. We were not able to find any infinite family of such matrices so that the smallest coefficient in any set of homology generating cycles grows exponentially with the size of *X* (that is, the size of the matrix). However, if a set of homology-generating cycles with subexponential coefficients always exists and can be found algorithmically in polynomial time, our main algorithm given as Theorem A is optimal in this case as well. This is because the exponential complexity of the algorithm only appears in the geometric realization of an element of $GX_1^{sph}$ with large (exponential) exponents (see “Arrow 3” in Sect. 6), and the only source of such exponents is the homology $H_1(AX)\simeq H_2(X)$.

## Effective Hurewicz inverse

Here, we will prove Theorem 1 by directly describing the algorithm proposed in Berger (1991) and analysing its running time.

### Definition 7

Let *G* be a simplicial group. Then the Moore complex ${\tilde{G}}$ is a (possibly non-abelian) chain complex defined by ${\tilde{G}}_i:=G_i\cap (\bigcap _{j>0} \ker {d_j})$ endowed with the differential $d_0: {\tilde{G}}_i\rightarrow {\tilde{G}}_{i-1}$.

It can be shown that $d_0 d_0=1$ in ${\tilde{G}}$ and that $\mathrm {Im}(d_0)$ is a normal subgroup of $\ker d_0$ so that the homology $H_*({\tilde{G}})$ is well defined.

### Definition 8

Let *F* be a 0-reduced simplicial set, *GF* the associated simplicial group from Definition 2, and ${\widetilde{GF}}$ its Moore complex. We define *AF* to be the Abelianization of *GF* and ${\widetilde{AF}}$ to be the Moore complex of *AF*. The simplicial group *AF* is also endowed with a chain group structure via $\partial = \sum _j (-1)^j d_j$. If $\sigma \in F_k$, we will denote by ${\overline{\sigma }}$ the corresponding simplex in $GF_{i-1}$, resp. $AF_{i-1}$.

Note that, following Definition 2, the “last” differential $d_k {\overline{\sigma }}$ in $AF_k$ equals $\overline{d_k \sigma } - \overline{d_{k+1}\sigma }$. Clearly, the Abelianization map $p: GF\rightarrow GF/[GF,GF]=AF$ takes ${\widetilde{GF}}$ into ${\widetilde{AF}}$.

Kan (1958a) showed that for $d>1$ and a $(d-1)$-connected simplicial set *F*, the Hurewicz isomorphism can be identified with the map $H_{d-1}({\widetilde{GF}})\rightarrow H_{d-1}({\widetilde{AF}})$ induced by Abelianization, whereas these groups are naturally isomorphic to $\pi _d(F)$ and $H_d(F)$, respectively. Our strategy is to construct maps representing the isomorphisms 1, 2, 3 in the commutative diagram3Here *h* stands for the Hurewicz isomorphism, 1 is induced by a homotopy equivalence of chain complexes, 2 is the inverse of $H_{d-1}(p)$ where *p* is the Abelianization, and 3 represents an isomorphism between the $(d-1)$’th homology of ${\widetilde{GF}}$ (that models the loop space of *F*) and $\pi _{d}(F)$. The algorithms that compute 1, 2, 3 act on representatives, that is, 1 and 2 map cycles to cycles and 3 converts a cycle to a simplicial map ${\varSigma }^d\rightarrow F$ where $|{\varSigma }^d|=S^d$. In what follows, we will explicitly describe maps 1, 2, 3 and show that the underlying algorithms are polynomial for arrows 1, 2 and exponential for arrow 3.

### Arrow 1

Let *F* be a 0-reduced simplicial set, $C_*(F)$ be the (unreduced) chain complex of *F* and $AF_{*-1}$ the shifted chain complex of *AF* defined by $(AF_{*-1})_i:=AF_{i-1}$. As a chain complex, $AF_{*-1}$ is a subcomplex of $C_*(F)$ generated by all simplices that are not in the image of the last degeneracy. Let ${\widetilde{AF}}_{*-1}$ be the Moore complex of $AF_{*-1}$.

We will describe a chain homotopy $(f,g,h): C_*(F) \rightarrow {\widetilde{AF}}_{*-1}$. Arrow 1 then coincides, on the level of chains, with *f*. We only need *f* for the actual algorithm; however, we prefer to state a more general Lemma claiming that *g*, *h* are polynomial time maps as well.

#### Lemma 8

There exists a polynomial-time strong chain deformation retraction $(f,g,h): C_*(F)\rightarrow {\widetilde{AF}}_{*-1}$. That is, $f: C_*(F)\rightarrow {\widetilde{AF}}_{*-1}$, $g: {\widetilde{AF}}_{*-1}\rightarrow C_*(F)$ are polynomial-time chain-maps and $h: C_*(F)\rightarrow C_{*+1}(F)$ is a polynomial map such that $fg=\mathrm {id}$ and $gf-\mathrm {id}=h\partial + \partial h$.

#### Proof

First we will describe the deformation retraction in terms of formulas and then comment on polynomiality.

*Part 1: Formulas for the deformation retraction.* We begin with a chain deformation retraction from $C_*(F)$ to $AF_{*-1}$ represented by $f_0: C_*(F)\rightarrow AF_{*-1}$, $g_0: AF_{*-1}\rightarrow C_*(F)$ and $h_0: C_*(F)\rightarrow C_{*+1}(F)$.

The chain complex $AF_{*-1}$ consists of Abelian groups $AF_{k-1}$ freely generated by *k*-simplices in *F* that are not in the image of the last degeneracy $s_{k-1}$. On generators, we define

4 $$\begin{aligned} f_0(\sigma ):= {\left\{ \begin{array}{ll} 0 \quad \text {whenever }\sigma \text { is a }k\text {-simplex in }\mathrm {Im}(s_{k-1})\\ {\overline{\sigma }} \quad \text {otherwise. } \end{array}\right. } \end{aligned}$$

The remaining maps are defined by $g_0({\overline{\sigma }}):=\sigma -s_{k-1} d_k \sigma$ and $h_0(\sigma ):=(-1)^k s_k \sigma$. It is a matter of straight-forward computations to check that $f_0$ and $g_0$ are chain maps, $f_0 g_0=\mathrm {id}$ and $g_0 f_0-\mathrm {id}=h_0\partial + \partial h_0$.

Further, we define another chain deformation retraction from *AF* to ${\widetilde{AF}}$. For each $p\ge 0$, let $A^p$ be a chain subcomplex of *AF* defined by

$$\begin{aligned} (A^p)_k:=\{x\in AF_{k}: d_i x=0\quad \text {for} \quad i>\max \{k-p,0\} \,\} \end{aligned}$$

that is, the kernel of the *p* last face operators, not including $d_0$ ($d_i$ refers here to the face operators in *AF*). Then $A^{p+1}$ is a chain subcomplex of $A^p$ and we define the maps $f_{p+1}: (A^p)_k\rightarrow (A^{p+1})_k$ by $f_{p+1}(x)=x-s_{k-p-1} d_{k-p} x$ whenever $k-p>0$, and $f_{p+1}(x)=x$ otherwise; $g_{p+1}: A^{p+1}\rightarrow A^p$ will be an inclusion, and $h_{p+1}: (A^p)_k\rightarrow (A^p)_{k+1}$ via $h_{p+1}(x)=(-1)^{k-p} s_{k-p}x$ if $k-p>0$ and 0 otherwise. A simple calculation shows that $f_{p+1}, g_{p+1}$ are chain maps, $f_{p+1} g_{p+1} = \mathrm {id}$, $g_{p+1} f_{p+1}-\mathrm {id}=h_{p+1}\partial + \partial h_{p+1}$.

By definition, the Moore complex ${\widetilde{AF}}=\cap _{p>0} A^p$. The strong chain deformation retraction (*f*, *g*, *h*) from $C_*(F)$ to ${\tilde{AF}}_{*-1}$ is then defined by the compositions

$$\begin{aligned}&f:=\ldots f_{k+1} f_k \ldots f_1 f_0 \\&g:=g_0 g_1 \ldots g_k g_{k+1} \ldots \end{aligned}$$

and the sum

$$\begin{aligned} h=h_0 + g_1 h_1 f_1 + (g_1 g_2) h_2 (f_2 f_1) + \cdots \end{aligned}$$

which are all well-defined, because when applying them to an element *x*, only finitely many of $f_j, g_j$ differ from the identity map and only finitely many $h_j$ are nonzero.

*Part 2: Polynomiality.* We need to show that if the degree *k* is fixed, then we can evaluate *f*, *g*, *h* on $C_k(F)$ resp. ${\widetilde{AF}}_{k-1}$ in time polynomial in the input size. The map $f_0$ is defined via the *if-else* condition (4). To decide whether a simplex $\sigma \in F(I)$ is in the image of $s_{k-1}$ amounts to deciding $\sigma =s_{k-1} d_k \sigma$ which can be done in time polynomial in $\mathrm {size}(I)+\mathrm {size}(\sigma )$, the polynomial depending on *k*. It follows that $f_0$ is a locally polynomial map. All the remaining maps $f_i, g_i$ and $h_i$ are defined via simple formulas and are obviously locally polynomial-time maps.

For fixed *k*, the definition of *f*, *g*, *h* includes only $f_i, g_i, h_i$ for $i<k$. It follows that *f*, *g* are composed of *k* polynomial-time maps and *h* is a sum of *k* polynomial-time maps. $\square$

### Arrow 2

This part is taken almost completely from Berger (1991), we only slightly adjusted the notation to our settings, formalized some details that in Berger (1991) are treated as obvious, and comment on polynomiality.

To summarize the main ideas, we will define an algorithm for computing *contraction* operators $GF_j \rightarrow GF_{j+1}$ that geometrically represent contraction of spheres in the loop space of *F*. The first such contraction $c_0: GF_0\rightarrow GF_1$ actually corresponds to the contraction of loops in *F* and cannot be derived algorithmically in general. That’s the reason why we insist on having some kind of information about the loop contraction $c_0$. Higher contractions, however, can be derived via formulas, assuming the input is $(d-1)$-connected (we don’t have a good intuition for this fact, but the Hurewicz isomorphism is probably the key; it is easy to construct these contractions on the Abelian part and the hard work is to pull them back to the non-commutative ${\widetilde{GF}}$). Formulas for the contractions $c_k$ are the core of Arrow 2.5Given an algorithm for the contractions, Arrow 2 is then defined as follows. For a cycle $z\in {\widetilde{AF}}_{k+1}$, we compute an arbitrary *p*-preimage *y*, $p(y)=z$, and then adjust it to $y \, (c_{k} d_0 y)^{-1}$ which already is a cycle in ${\widetilde{GF}}_{k+1}$.

We remark that without having something like the contraction data $c_k$, it is hard to find *any* non-trivial spherical elements of ${\widetilde{GF}}_*$.

#### Lemma 9

(Boundary certificate) Let $d>1$ be fixed and let *F* be a $(d-1)$-connected simplicial set with polynomial-time homology. There is an algorithm that, for $j<d-1$ and a cycle $z\in Z_{j}({\widetilde{AF}})$, computes an element $c^A(z)\in {\widetilde{AF}}_{j+1}$ such that $d_0 c^{A}(z)=z$. The running time is polynomial in $\mathrm {size}(z)+\mathrm {size}(I)$.

#### Proof

First note that the $(d-1)$-connectedness of *F* implies that $H_{j+1}(F)\simeq H_{j}({\widetilde{AF}})$ are trivial for $j<d-1$, so each cycle in these dimensions is a boundary.

By assumption, *F* has a polynomial-time homology, which means that there exists a globally polynomial-time chain complex $E_*F$, a locally polynomial-time chain complex *Y* and polynomial-time reductions from *Y* to $C_*(F)$ and $E_*F$Let $(f',g',h')$ be chain homotopy equivalence of *Y* and ${\widetilde{AF}}_{*-1}$ defined as the composition of  and the chain homotopy equivalence of $C_*(F)$ and ${\widetilde{AF}}_{*-1}$ described in Lemma 8. Further, let $f'', g'', h''$ be the maps defining the reduction : all of these maps are polynomial-time.

Let $j<d-1$ and $z\in Z_j({\widetilde{AF}})$, $z=\sum _j k_j y_j$. Then the element $f'' g'(z)$ is a cycle in $E_{j+1}F$ and can be computed in time polynomial in $\mathrm {size}(z)+\mathrm {size}(I)$. In particular, the size of $f''g'(z)$ is bounded by such polynomial. The number of generators of $E_{j+2}F$ and $E_{j+1}F$ is polynomial in $\mathrm {size}(I)$ and we can compute, in time polynomial in $\mathrm {size}(I)$, the boundary matrix of $\partial : E_{j+2}F\rightarrow E_{j+1}F$ with respect to the generators.

Next we want to find an element $t\in E_{j+2}F$ such that $\partial t=f''g'(z)$. Using generating sets for $E_{j+2}F$, $E_{j+1}F$, this reduces to a linear system of Diophantine equations and can be solved in time polynomial in the size of the $\partial$-matrix and the right hand side $f''g'(z)$ (Kannan and Bachem 1981).

Finally, we claim that $c^A(z):=f'g''(t)-f'h''g'(z)$ is the desired element mapped to *z* by the differential in ${\widetilde{AF}}$. This follows from a direct computation

$$\begin{aligned} \partial c^A(z)&:=\partial f'g''(t) - \partial f'h''g'(z) \\&=f'g''(\partial t)-\partial f'h'' g'(z) \\&= f'g''f''g'(z)-\partial f'h''g'(z) \\&= f'(h''\partial + \partial h''+\mathrm {id})g'(z)-\partial f'h''g'(z) \\&= f'h''g'\partial z + \partial f'h''g'(z) + f'g'(z) - \partial f'h''g'(z) \\&= 0 + f'g'(z) = z \end{aligned}$$

The computation of *t* as well as all involved maps are polynomial-time, hence the computation of $c^A(z)$ is polynomial too. $\square$

The next lemma will be needed as an auxiliary tool later.

#### Lemma 10

Let *S* be a countable set with a given encoding, *G* be the free (non-abelian) group generated by *S*, and define $\mathrm {size}(\prod _j s_j^{k_j}):=\sum _j (\mathrm {size}(s_j)+\mathrm {size}(k_j))$. Let $G':=[G,G]$ be its commutator subgroup.

Then there exists a polynomial-time algorithm that for an element $g=\prod _j s_j^{k_j}$ in $G'\subseteq G$, computes elements $a_i, b_i\in G$ such that $g=\prod _j [a_j, b_j]$.

In other words, we can decompose commutator elements into simple commutators in polynomial-time at most.

#### Proof

Let us choose a linear ordering on *S* and let $g=\prod _j s_j^{k_j}$ be in $G'$: that is, for each *j*, the exponents $\{k_{j'}:\,s_{j'}=s_j\}$ sum up to zero. We will present a bubble-sort type algorithm for sorting elements in *g*. Going from the left to right, we will always swap $s_j^{k_j}$ and $s_{j+1}^{k+1}$ whenever $s_{j+1}<s_j$. Such swap always creates a commutator, but that will immediately be moved to the initial segment of commutators.

More precisely, assume that Init is the initial segment, $x=s_j^{k_j}$ and $y=s_{j+1}^{k_{j+1}}$ should be swapped, Rest represent the segment behind *y*, and Commutators is a final segment of commutators. The swapping will consists of these steps:

$$\begin{aligned}&\text {Init} \,\, x \,\, y \,\, \text {Rest} \,\, \text {Commutators} \\&\quad \mapsto \text {Init} \,\, y \,\, x \,\, [x^{-1}, y^{-1}] \,\, \text {Rest} \,\,\text {Commutators} \\&\quad \mapsto \text {Init} \,\, y \,\, x \,\, \text {Rest} \,\, \big ([x^{-1}, y^{-1}] \,\, [[y^{-1}, x^{-1}], \text {Rest}^{-1}] \,\, \text {Commutators} \big ) \end{aligned}$$

where the parenthesis enclose a new segment of commutators. Before the parenthesis, *x* and *y* are swapped. Each such swap requires enhancing the commutator section by two new commutators of size at most $\mathrm {size}(g)$, hence each such swap has complexity linear in $\mathrm {size}(g)$.

Let us call everything before the commutator section a “regular section”. Going from left to right and performing these swaps will ensure that the largest element will be at the end of the regular section. But no later then that, the largest element $y_{\text {largest}}$ disappears from the regular section completely, because all of its exponents add up to 0. Again, starting from the left and performing another round of swaps will ensure that the second-largest elements disappear from the regular section; repeating this, all the regular section will eventually disappear which will happen in at most $\mathrm {size}(g)^2$ swaps in total. Each swap has complexity linear in $\mathrm {size}(g)$ and the overall time complexity is not worse than cubic. $\square$

#### Lemma 11

Assume that *F* is a parametrized simplicial set with polynomially contractible loops. Let $k>0$, $\gamma \in GF_k$ be spherical, i.e. $d_i \gamma = 1$, $0\le i\le k$, and $\alpha \in GF_k$ is arbitrary. There is a polynomial-time algorithm that computes $\delta \in GF_{k+1}'$ such that $d_0 \delta =[\alpha , \gamma ]$ and $d_i \delta =1$ for all $i>0$.

In other words, a simple commutator of a spherical element with another element can always be “contracted” in $GF'$ in polynomial time. Our proof roughly follows the construction in Kan (1958a, Sect. 8).

#### Proof

For $x\in GF_0$, we will denote by $c_0 x$ the element of ${\widetilde{GF}}_1$ such that $d_0 c_0 x=x$: this can be computed in polynomial-time by the assumption on polynomial loop contractions. For the simplex $\alpha \in GF_k$, we define $(k+1)$-simplices $\beta _0,\ldots , \beta _k$ by $\beta _k:=s_0^k c_0 d_0^k \alpha$ and inductively $\beta _{j-1}:=(s_j d_j \beta _j) \,\cdot \, (s_j \alpha ^{-1})\,\cdot \,(s_{j-1} \alpha )$ for $j<k$. Then the following relations hold:^13^

- $$d_0 \beta _0=\alpha $$.
- $$d_j \beta _j=d_j \beta _{j-1}$$, $$1\le j\le k$$
- $$d_{k+1} \beta _k=1$$.

The second and third equations are a matter of direct computation, while the first follows from the more general relation $d_0^{j+1} \beta _j=d_0^j \alpha$ which can be proved by induction. If *k* is fixed, then all $\beta _0,\ldots ,\beta _k$ can be computed in polynomial time.

The desired element $\delta$ is then the alternating product

$$\begin{aligned} \delta :=[\beta _0, s_0 \gamma ] \, [\beta _1, s_1\gamma ]^{-1} \, \ldots \,[\beta _k, s_k\gamma ]^{\pm 1}.\qquad \qquad \qquad \qquad \qquad \qquad \qquad \qquad \qquad \qquad \square \end{aligned}$$

#### Lemma 12

Under the assumptions of Theorem 1, there exist homomorphisms $c_j: {GF}_j\rightarrow {GF}_{j+1}$ for $0\le j < d-1$ such that

1. $$d_0 c_j=\mathrm {id}$$,
2. $$d_i c_j=c_{j-1}d_{i-1}$$, $$0<i\le j+1$$, and
3. $$c_{j} s_{i} = s_{i+1} c_{j-1}$$ for $$0<j<d-1$$ and $$0\le i< j$$,
4. $$d_1 c_0(x)=1$$ for all $$x\in GF_0$$.

If *d* is fixed and $x\in GF_j$, $j<d-1$, then $c_j(x)$ can be computed in polynomial time.

#### Proof

The homomorphism $c_0$ can be constructed directly from the assumption on polynomial contractibility of loops. We have a canonical basis of $GF_0$ consisting of all non-degenerate 1-simplices of *F*. For $\sigma \in F_1$, we denote by ${\overline{\sigma }}$ the corresponding generator of $GF_0$. The we define $c_0(\prod {\overline{\sigma }}_j^{k_j})$ to be $\prod b_j^{k_j}$ where $b_j$ is the element of $GF_1$ such that $d_0 b_j={\overline{\sigma }}_j$ and $d_1 b_j=1$.

In what follows, assume that $1\le k<d-1$ and $c_i$ have been defined for all $i<k$. We will define $c_k$ in the following steps.

**Step 1.** Contractible elements.

Let $x\in GF_k$. We will say that *x* is *contractible* and $y\in GF_{k+1}$ is a *contraction of x* if $d_0 y=x$ and $d_i y=c_{k-1} d_{i-1} x$ for all $i>0$.

The general strategy for defining $c_k$ will be to find a contraction *h* for each basis element ($(k+1)$-simplex) $g\in GF_k$ and define $c_k(g):=h$. This will enforce properties 1 and 2. Moreover, in case when *g* is degenerate, the contraction will be chosen in such a way that property 3 holds too; otherwise it holds vacuously. Property 4 only deals with $c_0$ and is satisfied by the definition of loop contraction (a polynomial-time $c_0$ is given as an input in Theorem 1).

**Step 2.** Contraction of degenerate elements.

Let $g=s_i y$ be a basis element of $GF_k$, $y\in GF_{k-1}$. Then *g* can be uniquely expressed as $s_{j} z$ where *j* is the maximal *i* such that $g\in \mathrm {Im}(s_i)$. We then define $c_k(g):=s_{j+1} c_{k-1}(z)$. Note that

$$\begin{aligned} d_0 c_k(g)=d_0 s_{j+1} c_{k-1}(z)=s_j d_0 c_{k-1}(z)=s_j z=g, \end{aligned}$$

so property 1 is satisfied. To verify property 2, first assume that $i\in \{j+1, j+2\}$. Then we have

$$\begin{aligned} d_i c_k(g)= d_i s_{j+1} c_{k-1} (z)=c_{k-1} (z)=c_{k-1} d_{i-1} s_{j} z=c_{k-1} d_{i-1} g. \end{aligned}$$

This fully covers the case $k=1$, because then the only possibility is $j=0$ and $i\in \{1,2\}$. Further, let $k>1$. If $i\le j$, then we have

$$\begin{aligned} d_i c_k g&= d_i c_k s_j z=d_i s_{j+1} c_{k-1}(z)=s_j d_i c_{k-1}(z) = s_j c_{k-2} d_{i-1} z\\&=c_{k-1} s_{j-1} d_{i-1} z =c_{k-1} d_{i-1} s_j z=c_{k-1} d_{i-1} g \end{aligned}$$

and if $i>j+2$, then the computation is completely analogous, using the relation $d_i s_{j+1}=s_{j+1} d_{i-1}$ instead.

So far, we have shown that $c_k(g):=s_{j+1} c_{k-1} g$ is a contraction of *g*. It remains to show property 3. That is, we have to show that if $g=s_j z$ can also be expressed as $s_i y$, then $c_k(s_i y)=s_{i+1} c_{k-1} y$.

The degenerate element *g* has a unique expression $g=s_{i_u} \ldots s_{i_1} s_{i_0} v$ where $i_0<i_1<\cdots <i_u=j$ and is expressible as $s_i x$ iff $i=i_j$ for some $j=0,1,\ldots , u$. Choosing such $i<j$, we can rewrite *g* as $g=s_j s_i v$ for some *v* and then $g=s_i s_{j-1} v$, so that $y=s_{j-1} v$ and $z=s_i v$. Then we again use induction to show

$$\begin{aligned} c_k(s_i y)&=s_{j+1} c_{k-1}(z)=s_{j+1} c_{k-1} s_i v=s_{j+1} s_{i+1} c_{k-2} v \\&= s_{i+1} s_j c_{k-2} v=s_{i+1} c_{k-1} s_{j-1} v=s_{i+1} c_{k-1} y \end{aligned}$$

as required.

**Step 3.** Decomposition into spherical and conical parts.

We will call an element ${\hat{x}}\in GF_k$ to be *conical* if it is a product of elements that are either degenerate or in the image of $c_{k-1}$. Let $x\in GF_k$ be arbitrary. We define $x_k:=x$ and inductively $x_{i-1}:=x_i (s_{i-1} d_i x_i)^{-1}$. In this way we obtain $x_0,\ldots , x_n$ such that $x_i$ is in the kernel of $d_j$ for $j>i$ and $x=x_0 y$ where *y* is a product of degenerate simplices. Further, let $x^s:=x_0 (c_{k-1} d_0 x_0)^{-1}$. A simple computation shows that $x^s$ is *spherical*, that is, $d_i x^s=1$ for all *i*. We obtain an equation $x=x^s {\hat{x}}$ where ${\hat{x}}=(c_{k-1} (d_0 x_0) y$; this is a decomposition of *x* into a spherical part $x^s$ and a conical element ${\hat{x}}$.

We will define $c_k$ on non-degenerate basis elements $g={\overline{\sigma }}$ by first decomposing $g=g^S {\hat{g}}$ into a spherical and conical part, finding contractions $h_1$ of $g^S$ and $h_2$ of ${\hat{g}}$, and defining $c_k(g):=h_1 h_2$. Then $c_k(g)$ is a contraction of *g* and hence satisfies properties 1 and 2: property 3 is vacuously true once *g* is non-degenerate.

**Step 4.** Contraction of the conical part.

Let ${\hat{x}}:=c_{k-1} (d_0 x_0)\, y$ be the conical part defined in the previous step. By construction, $x_0\in {\tilde{GF}}_k$ and *y* is a product of degenerate elements $s_{i_1} u_1 \ldots s_{i_l} u_l$. We define the contraction of $c_{k-1} (d_0 x_0)$ to be

$$\begin{aligned} {\tilde{c}}_{k}(c_{k-1}(d_0 x_0)):=s_0 c_{k-1}(d_0 x_0). \end{aligned}$$

Note that this satisfies property 1 as $d_0 {\tilde{c}}_k c_{k-1} (d_0 x_0)=c_{k-1} (d_0 x_0)$. For property 2, we first verify

$$\begin{aligned} d_1 {\tilde{c}}_k c_{k-1} (d_0 x_0)=d_1 s_0 c_{k-1}(d_0 x_0)=c_{k-1}(d_0 x_0)=c_{k-1} d_0 c_{k-1} (d_0 x_0). \end{aligned}$$

Not let $i\ge 2$. If $k=1$, then the remaining face operator is $d_2$ and we have

$$\begin{aligned} d_2 {\tilde{c}}_1 c_0 (d_0 x_0)=d_2 s_0 c_0(d_0 x_0)=s_0 d_1 c_0(d_0 x_0)=1=c_0 d_1 c_0(d_0 x_0) \end{aligned}$$

using axiom 4 for $c_0$. Finally, if $i\ge 2$ and $k\ge 2$, we have

$$\begin{aligned} d_i {\tilde{c}}_k c_{k-1} (d_0 x_0)&=d_i s_0 c_{k-1}(d_0 x_0)=s_0 d_{i-1} c_{k-1} (d_0 x_0)=s_0 c_{k-1} d_{i-2} d_0 x_0 \\&= s_0 c_{k-1} d_0 d_{i-1} x_0=s_0 c_{k-1} d_0 1= 1 = c_{k-1} c_{k-2} d_0 d_{i-1} x_0\\&= c_{k-1} c_{k-2} d_{i-2} d_0 x_0=c_{k-1} d_{i-1} c_{k-1}(d_0 x_0), \end{aligned}$$

where we exploited the fact that $x_0\in {\widetilde{GF}}_k$ and hence $d_{u} x_0=1$ for $u\ge 2$.

The contraction of degenerate elements *y* has already been defined in Step 2, so we can define a contraction of $c_{k-1}(d_0 x_0) y$ to be $s_0 c_{k-1}(d_0 x_0)\,c_k(y)$.

**Step 5.** Contraction of commutators.

Let $g'\in GF_k'$ be an element of the commutator subgroup. By Lemma 10, we can algorithmically decompose $g'$ into a product of simple commutators, so to find a contraction of $g'$, it is sufficient to find a contraction of each simple commutator [*x*, *y*] in this decomposition.

Let $x=x^S\,{\hat{x}}$ and $y=y^S\,{\hat{y}}$ be the decompositions into spherical and conical parts described in Step 3. Using the notation $^b a:=bab^{-1}$, we can decompose [*x*, *y*] as follows (Berger 1991, p. 60):

6 $$\begin{aligned}{}[x,y]=\,([x,y] [{\hat{y}}, x])\,([x,{\hat{y}}] [{\hat{y}}, {\hat{x}}]) \,[{\hat{x}}, {\hat{y}}] = [^{xy} x^{-1}, ^{xy}(y^{-1} {\hat{y}})] \,[^x{\hat{y}}, ^x(x^{-1}{\hat{x}})]\,[{\hat{x}},{\hat{y}}]. \end{aligned}$$

Both $x^{-1}{\hat{x}}$ and $y^{-1}{\hat{y}}$ are spherical simplices and so are their conjugations. It follows that Eq. (6) can be rewritten to $[x,y]=[\alpha _1, \gamma _1]\,[\alpha _2,\gamma _2]\,[{\hat{x}}, {\hat{y}}]$ where $\gamma _{1}$ and $\gamma _2$ are spherical. All of these decompositions are done by elementary formulas and are polynomial-time in the size of *x* and *y*.

By Lemma 11 we can find an elements $\lambda _i\in {\widetilde{GF}}_{k+1}$ such that $d_0 \lambda _i=[\alpha _i,\gamma _i]$, $i=1,2$, in polynomial time. Further, both ${\tilde{x}}$ and ${\tilde{y}}$ are conical and they are in the form ${\tilde{x}}=c_0 (d_0 x_0) x_{deg}$ where $x_0\in {\widetilde{GF}}_k$ and $x_{deg}$ is degenerate; similar decomposition holds for *y*. In Step 4 we showed how to compute elements $c^x$ and $c^y$ such that $c^x$, $c^y$ is a contraction of ${\hat{x}}$, ${\hat{y}}$, respectively. Then $[c^x, c^y]$ is a contraction of $[{\hat{x}}, {\hat{y}}]$ and $\lambda _1 \lambda _2 [c^x, c^y]$ is a contraction of [*x*, *y*].

**Step 6.** Contraction of spherical elements.

The last missing step is to compute a contraction of the spherical element $g^S$ where $g^S$ is the spherical part of a basis element $g\in GF_k$.

Let us denote by *p* the projection $GF{\mathop {\rightarrow }\limits ^{p}} AF$. The projection $z:=p(g^S)$ is in the kernel of all face operators and hence a cycle in ${\widetilde{AF}}_{k}$. By Lemma 9, we can compute $t:=c_k^A(z)\in {\widetilde{AF}}_{k+1}$ such that $d_0 t=z$, in polynomial time. Let $h\in {GF}_{k+1}$ be any *p*-preimage^14^ of *t*. Let $h_k:=h$ and inductively define $h_{j-1}:=h_{j} (s_{j-1} d_j h_{j})^{-1}$ for $j<k$. Then $h_0$ is in the kernel of all faces except $d_0$, that is, $h_0\in {\widetilde{GF}}_{k+1}$. It follows that $p(h_0)\in {\widetilde{AF}}_{k+1}$ is in the kernel of all faces except $d_0$. We claim that $p(h_0)=t$.This can be shown as follows: assume that $p(h_j)=t$, then $p(h_{j-1})=p(h_j) + p(s_{j-1} d_j h_j^{-1})=t + s_{j-1} d_j t=t+0=t$.

We have the following commutative diagram:Both $$g^S$$ and $$d_0 h_0$$ are mapped by *p* to the same element *z*: it follows that $$g^S (d_0 h_0)^{-1}$$ is mapped by *p* to zero and hence is an element of the commutator subgroup. Let $${\tilde{h}}$$ be the contraction of $$g^S (d_0 h_0)^{-1} $$, computed in Step 5, and finally let $$h:={\tilde{h}} h_0$$. Then *h* is an element of $${\widetilde{GF}}_{k+1}$$ and a direct computation shows that $$d_0 {h}=g^S$$ as desired.

This completes the construction of $c_k$: for each non-degenerate basis element *g* of $GF_k$, $c_k(g)$ is defined to be the product of the contraction of $g^S$ and the contraction^15^ of ${\hat{g}}$.

All the subroutines described in the above steps are polynomial-time. Thus we showed that if there exists a polynomial-time algorithm for $c_{k-1}$, then there also exists a polynomial-time algorithm for $c_k$. The existence of a polynomial-time $c_0$ follows from the assumption on polynomial loop contractibility and *d* is fixed, thus there exists a polynomial-time algorithm that for $x\in GF_{j}$ computes $c_j(x)$ for each $j<d-1$. $\square$

#### Lemma 13

(Construction of arrow 2) Under the assumption of Theorem 1, let $z\in Z_{d-1}({\widetilde{AF}})$ be a cycle. Then there exists a polynomial-time algorithm that computes a cycle $x\in Z_{d-1}({\widetilde{GF}})$ such that the Abelianization of *x* is *z*.

The assignment $z\mapsto x$ is hence an effective inverse of the isomorphism

$$\begin{aligned} H_{d-1}({\widetilde{GF}})\rightarrow H_{d-1}({\widetilde{AF}}) \end{aligned}$$

on the level of representatives.

#### Proof

Let $c_{d-2}$ be the contraction from Lemma 12 and $z\in Z_{d-1}({\widetilde{AF}})$ be a cycle. First choose $y\in GF_{d-1}$ such that $p(y)=z$. Creating the sequence $y_n:=y$, $y_{j-1}:=y_j s_{j-1} d_j y_j^{-1}$ for decreasing *j*, yields an element $y_0\in {\widetilde{GF}}_{d-1}$ that is still mapped to *z* by *p*, similarly as in Step 4 of Lemma 12. The equation $p d_0 (y_0)=d_0 p (y_0)=d_0 z=0$ shows that $d_0 y_0$ is in the commutator subgroup ${\widetilde{GF}}_{d-2}'$. We define $x:=y_0 c_{d-2} (d_0 y_0)^{-1}$: this is already a cycle in ${\widetilde{GF}}_{d-1}$ and $p(x)=p(y_0)=z$. $\square$

### Arrow 3

A cycle $g \in {\widetilde{GF}}_{d-1}\subseteq GF_{d-1}$ represents a generator of the homotopy group $H_{d-1} ({\widetilde{GF}}) \cong \pi _{d} (F)$. Given such *g*, our goal is to construct a simplicial set ${\varSigma }^{d}$ with $|{\varSigma }^{d}| = S^{d}$ and a simplicial map $\gamma _g: {\varSigma }^{d} \rightarrow F$ with $[\gamma _g] \cong [g]$.

**Fig. 3:**
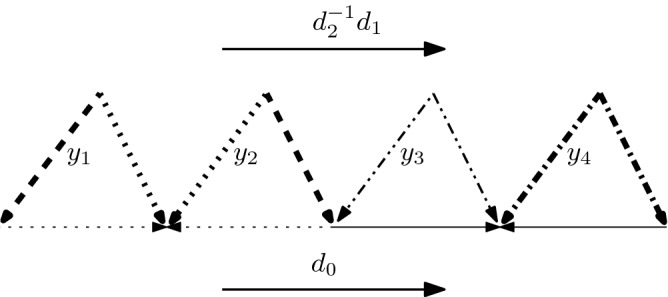
Let $g\in GF_1$ with $g = \overline{x_1 x_2 x_3 x_4}$, where $d_0 (x_1) = d_0 (x_2)^{-1}$, $d_0 (x_3) = d_0 (x_4)^{-1}$, $d_2 (x_1) = d_1 (x_2)$, $d_2 (x_2) = d_1 (x_1)$, $d_1 (x_3) = d_2 (x_3)$ and $d_1 (x_4) = d_2 (x_4)$. Simplicial set *Y* is then obtained by identifying faces of triangles $y_1, y_2, y_3, y_4$ as pictured here. One can see $|Y| = S^2 \vee S^2$

Consider first the following naive idea of the construction: Suppose that $g= \overline{x_1 \cdots x_n}$, where $x_i \in F_d, 1\le i \le n$. For simplicity, assume that $x_i$’s are all nondegenerate simplices. We then take *n* disjoint simplices $y_1, \ldots y_n$ and define $f: \bigsqcup _{i = 1}^{n} y_i \rightarrow F$ sending $y_i$ to $x_i$. From the fact that $d_j (\overline{x_1 \cdots x_n}) = 1$, we now define relations on the faces of $y_i$’s (say we conclude that $d_j (x_i) = d_j(x_{i+1})^{-1}$, then we add relation $d_j (y_i) \sim d_j (y_{i+1})$). Putting all such relations together we define a simplicial set $Y = \bigsqcup _{i = 1}^{n} y_i / \sim$ and a simplicial map $f': Y \rightarrow F$ where *f* factors through $f'$, see Fig. 3 for an example.

We have nearly achieved what we aimed for - one can show that |*Y*| is homotopy equivalent to the wedge sum of *d* - dimensional spheres and $[f']\cong g$. However, in general |*Y*| is not homeomorphic to $S^d$. One way to overcome the problem is to make the space *Y* “thicker” and making sure that identifying simplices never results in a wedge of spheres.

To this end, we utilize the following construction which is one of the main results from Berger (1995). Here, we describe the main points of the construction while details are given in later sections.

On an algebraic level, we define another simplicial model of a loopspace of *F*—a simplicial group ${\overline{{\varOmega }}}F$. Further, there is a homomorphism of simplicial groups $t:GF \rightarrow {\overline{{\varOmega }}}F$ that induces an isomorphism on the level of homotopy groups. This is described in Berger (1995, Proposition 3.3).

The homomorphism *t* is given later by formula (8) and the simplicial set ${\overline{{\varOmega }}}F$ is described in the next section. Here, we remark that the size of *t*(*g*) is exponential in size of *g*.

Finally, Lemma 14 describes an algorithm that for a spherical element $\gamma \in {\overline{{\varOmega }}}F_{d-1}$ constructs a simplicial map $\gamma _\mathsf {sph}: {\varSigma }^{d}(\gamma ) \rightarrow F$ such that $\pi _{d-1}({\overline{{\varOmega }}}F)\ni [\gamma ] \simeq [\gamma _\mathsf {sph}] \in \pi _{d}(F)$ - essentially using the naive idea described above. We describe the construction in detail in the next section.

The size of $\gamma _\mathsf {sph}$ is polynomial in $\mathrm {size}(\gamma )$. Hence, given a spherical $g \in {\widetilde{GF}}_{d-1}$, the algorithm produces $t(g)_\mathsf {sph}:{\varSigma }^{d}(t(g)) \rightarrow F$ that is exponential with respect to $\mathrm {size}(g)$.

### Berger’s model of the loop space

#### Definition 9

(*Oriented multigraph on*$X_n$) Let *X* be a 0-reduced simplicial set. We define a directed multigraph $MX_n = (V_n,E_n)$, where the set of vertices $V_n = X_n$ and the set of edges $E_n$ is given by

$$\begin{aligned} E_n = \{[x, i]^\epsilon \mid x\in X_{n+1}, 0\le i \le n, \epsilon \in \{1, -1\}\}. \end{aligned}$$

We define maps $\mathsf {source} \,, \mathsf {target} \,: E_n \rightarrow V_{n}$ by setting $\mathsf {source} \,[x,i] = d_{i+1} x$, $\mathsf {target} \,[x,i] = d_i x$ and $\mathsf {source} \,[x,i]^{-1} = \mathsf {target} \,[x,i]$ and $\mathsf {target} \,[x,i]^{-1} = \mathsf {source} \,[x,i]$.

An edge $[x, i]^\epsilon \in E_n$ is called *compressible* if $x = s_i x'$ for some $x' \in X_{n}$.

#### Definition 10

(*Paths*) Let $X \in \mathsf {sSet}$. A sequence of edges in $MX_{n}$

7 $$\begin{aligned} \gamma = [x_1,i_1]^{\epsilon _1}[x_2,i_2]^{\epsilon _2}\cdots [x_k,i_k]^{\epsilon _k} \end{aligned}$$

is called an *n*-*path* if $\mathsf {target} \,[x_j,i_j]^{\epsilon _j} = \mathsf {source} \,[x_{j+1},i_{j+1}]^{\epsilon _{j+1}}$, $1\le j<k$.

Moreover, for every $x\in V_n = X_n$ we define a path of length zero $1_x$ with the property $\mathsf {source} \,1_x = x = \mathsf {target} \,1_x$ and relations $a 1_x=a$ whenever $\mathsf {target} \,{a}=x$ and $1_x b=b$ whenever $\mathsf {source} \,{b}=x$.

The set of paths on $MX_{n}$ is denoted by $IX_n$. Let $\gamma \in IX_n$ by as in (7). We define $\mathsf {source} \,\gamma = \mathsf {source} \,[x_1,i_1]^{\epsilon _1}$ and $\mathsf {target} \,\gamma =\mathsf {target} \,[x_k,i_k]^{\epsilon _k}$. The *inverse* of $\gamma$, denoted $\gamma ^{-1}$, is defined as

$$\begin{aligned} \gamma ^{-1} = [x_k,i_k]^{- \epsilon _k}\cdots [x_1,i_1]^{ - \epsilon _1}. \end{aligned}$$

if $\gamma$ = $1_x$, then $\gamma ^{-1} = \gamma$. Note that each path is either equal to $1_x$ for some *x* or can be represented in a form such as (7), without any units.

For algorithmic purposes, we assume that a path $\gamma = [x_1,i_1]^{\epsilon _1}[x_2,i_2]^{\epsilon _2}\cdots [x_k,i_k]^{\epsilon _k}$ is represented as a list of triples $(x_j, i_j, \epsilon _j)$ and has size

$$\begin{aligned} \mathrm {size}(\gamma ):=\sum _j \mathrm {size}(x_j)+\mathrm {size}(i_j)+\mathrm {size}(\epsilon _j), \end{aligned}$$

which is bounded by a linear function in $\sum _j \mathrm {size}({x_j})$.

Given an edge $[x,i]^\epsilon \in MX_n$, we define operators

$$\begin{aligned} d_0, \ldots d_{n}: E_n \rightarrow IX_{n-1} \,\, \text {and}\,\, s_0, \ldots , s_n: E_n \rightarrow IX_{n+1} \end{aligned}$$

called *face* and *degeneracy* operators, respectively. These are given as follows

$$\begin{aligned} d_j [x,i]^\epsilon = \left\{ \begin{array}{ll} {[}d_j x, i - 1{]}^\epsilon ,&{} \quad j< i;\\ 1_{d_ i d_{i+1} x},&{} \quad i=j;\\ {[}d_{j+1} x, i{]}^\epsilon ,&{} \quad j>i. \end{array} \right. \qquad s_j [x,i]^\epsilon = \left\{ \begin{array}{ll} {[}s_j x, i + 1{]}^\epsilon ,&{} \quad j< i;\\ {[}s_i x, i+1{]}{[}s_{i+1} x, i{]})^\epsilon ,&{} \quad i=j;\\ {[}s_{j+1} x, i{]}^\epsilon ,&{} \quad j>i. \end{array} \right. \end{aligned}$$

One can now extend the definition of face and degeneracy operators to paths, i.e. we define operators $d_0, \ldots d_{n}: IX_n \rightarrow IX_{n-1}$ and $s_0, \ldots , s_n: IX_n \rightarrow IX_{n+1}$

$$\begin{aligned} d_j \gamma= & {} \left\{ \begin{array}{ll} d_j([x_1,i_1]^{\epsilon _1}) d_j([x_2,i_2]^{\epsilon _2})\cdots d_j([x_k,i_k]^{\epsilon _k})&{}\quad \text {if} \; \gamma = [x_1,i_1]^{\epsilon _1}[x_2,i_2]^{\epsilon _2}\cdots [x_k,i_k]^{\epsilon _k},\\ 1_{d_j x}&{}\quad \text {if} \; \gamma = 1_x, x\in X_n. \end{array} \right. \\ s_j \gamma= & {} \left\{ \begin{array}{ll} s_j([x_1,i_1]^{\epsilon _1}) s_j([x_2,i_2]^{\epsilon _2})\cdots s_j([x_k,i_k]^{\epsilon _k})&{}\quad \text {if} \; \gamma = [x_1,i_1]^{\epsilon _1}[x_2,i_2]^{\epsilon _2}\cdots [x_k,i_k]^{\epsilon _k}\\ 1_{s_j x}&{}\quad \text {if} \; \gamma = 1_x, x\in X_n. \end{array} \right. \end{aligned}$$

With the operators defined above, one can see that *IX* is in fact a simplicial set.

For any $\gamma , \gamma ' \in IX$ such that $\mathsf {target} \,{\gamma } = \mathsf {source} \,{\gamma '}$, we define a composition $\gamma \cdot \gamma '$ in an obvious way.

If the simplicial set *X* is 0-reduced, we denote the unique basepoint $*\in X_0$. Abusing the notation, we denote the iterated degeneracy of the basepoint $\underbrace{{s_0} \cdots s_0 *}_{k\text {-times}}\in X_{k}$ by $*$ as well. With that in mind, we define simplicial subsets *PX*, ${\varOmega } X$ of *IX* as follows:

$$\begin{aligned} PX= \{\gamma \in IX \mid \mathsf {target} \,\gamma = *\} \quad {\varOmega } X= \{\gamma \in IX \mid \mathsf {source} \,\gamma = *= \mathsf {target} \,\gamma \}. \end{aligned}$$

We remark that simplicial sets $PX, {\varOmega } X$ intuitively capture the idea of path space and loop space in a simplicial setting.

#### Definition 11

A path $\gamma = [x_1,i_1]^{\epsilon _1}[x_2,i_2]^{\epsilon _2}\cdots [x_k,i_k]^{\epsilon _k} \in IX$ is called *reduced* if for every $1\le j<k$ the following condition holds:

$$ \begin{aligned} (x_j = x_{j+1}\, \& \,i_j = i_{j+1}) \Rightarrow \epsilon _j = \epsilon _{j+1}. \end{aligned}$$

e.g. an edge in the path $\gamma$ is never followed by its inverse.

An edge $[x, i]^\epsilon \in E_n$ is called *compressible* if $x = s_i x'$ for some $x' \in X_{n}$. A path is *compressed* if it does not contain any compressible edge.

We define relation $\sim _R$ on *IX* (or rather on each $IX_n$) as a relation generated by

$$\begin{aligned} {[}x,i]^{\epsilon } [x,i]^{-\epsilon } \sim _R 1_{\mathsf {source} \,( [x,i]^{\epsilon })},\quad n\in {\mathbb {N}}_0, [x,i]^\epsilon \in E_n.\end{aligned}$$

Similarly, we define $\sim _C$ on *IX* as a relation generated by

$$\begin{aligned}{}[x,i]^{\epsilon } \sim _C 1_{\mathsf {source} \,( [x,i]^{\epsilon })},\quad \text {if}\, [x,i]^{\epsilon } \in E_n \,\text {is compressible}. \end{aligned}$$

We finally define ${\overline{I}}X = (IX/\sim _C)/ \sim _R$. Similarly, one defines ${\overline{P}}X, {\overline{{\varOmega }}}X$.

For $\gamma , \gamma '\in IX_n$, we write $\gamma \sim \gamma '$ if they represent the same element in ${\overline{I}}X_n$. The symbol ${\overline{\gamma }}$, denotes the (unique) compressed and reduced path such that $\gamma \sim {\overline{\gamma }}$. One can see ${\overline{I}}X$ (${\overline{P}}X, {\overline{{\varOmega }}}X$) as the set of reduced and compressed paths in $IX (P X, {\varOmega } X)$.

In a natural way, we can extend the definition of face and degeneracy operators $d_i, s_i$ on sets ${\overline{I}}X ({\overline{P}}X$,${\overline{{\varOmega }}}X$) by setting $d_i \gamma = \overline{d_i \gamma }$ and $s_i \gamma = \overline{s_i \gamma }$. One can check that this turns ${\overline{I}}X$, ${\overline{P}}X$ and ${\overline{{\varOmega }}}X$ into simplicial sets.

Similarly, we define operation $\cdot : {\overline{{\varOmega }}}X_n \times {\overline{{\varOmega }}}X_n \rightarrow {\overline{{\varOmega }}}X_n$ by $\gamma \cdot \gamma ' \mapsto \overline{\gamma \gamma '}$, i.e. we first compose the loops and then assign the appropriate compressed and reduced representative. With the operation defined as above, ${\overline{{\varOmega }}}X$ is a simplicial group.

### Homomorphism ${\varvec{t: GX \rightarrow {\overline{{\varOmega }}}X}}$

We first describe how to any given $x \in X_n$ assign a path $\gamma _x \in {\overline{P}}X_n$ with the property $\mathsf {source} \,\gamma _x = x$ and $\mathsf {target} \,\gamma _x = *$:

For $x \in X_n$, $n>0$, the 0-reducedness of *X* gives us $d_{i_1} d_{i_2}\cdots d_{i_{n}} x = *$, here $i_{j} \in \{0, \ldots , j\}$, $0<j\le n$. In particular, $d_{0} d_{1}\cdots d_{n-1} x = *$. Using this, we define

$$\begin{aligned} \gamma _x = [s_{n}x,{n-1}][s_{n}s_{n-1}d_{n-1}x,{n-2}]\cdots [s_{n}s_{n-1}\cdots s_{1}d_1 d_{2}\cdots d_{n-1}x,0]. \end{aligned}$$

Ignoring the degeneracies, one can see the sequence of edges as a path

$$\begin{aligned} x \rightarrow d_{n-1}x \rightarrow d_{n-2}d_{n-1}x \rightarrow \cdots \rightarrow d_{0} d_{1}\cdots d_{n-1} x. \end{aligned}$$

We define the homomorphism *t* on the generators of $GX_n$, i.e. on the elements ${\overline{x}}$, where $x \in X_{n+1}$ as follows:

8 $$\begin{aligned} t({{\overline{x}}}) = \overline{\gamma _{d_{n+1}x}^{-1}[x,n]\gamma _{d_{n}x}}. \end{aligned}$$

This is an element of ${\overline{{\varOmega }}}X_n$.

The algorithm representing the map *t* has *exponential time complexity* due to the fact that an element ${\overline{\sigma }}^{k}$ with size $\mathrm {size}(\sigma )+\mathrm {size}(k)$ is mapped to

$$\begin{aligned} \underbrace{\overline{\gamma _{d_{n+1}x}^{-1}[x,n]\gamma _{d_{n}x} \,\,\, \ldots \,\,\,\gamma _{d_{n+1}x}^{-1}[x,n]\gamma _{d_{n}x}}}_{k \,\,\text {times}} \end{aligned}$$

which in general can have size proportional to *k*. Assuming an encoding of integers such that $\mathrm {size}(k)\simeq \ln (k)$, this amounts to an exponential increase.

### Universal preimage of a path

Intuitively, one can think of the simplicial set *IX* of paths as of a discretized version of space of continuous maps $|X|^{[0,1]}$. In particular, $\gamma \in IX_{d-1}$ is a walk through a sequence of *d*-simplices in *X* that connect $\mathsf {source} \,\gamma$ with $\mathsf {target} \,\gamma$. However, in the continuous case an element $\mu \in |X|^{[0,1]}$ corresponds to a continuous map $\mu : [0,1] \rightarrow |X|$. We want to push the parallels further, namely, given any nontrivial^16^$\gamma \in IX_{d-1}$, we aim to define a simplicial set $\mathsf {Dom}(\gamma )$ and a simplicial map $\gamma _\mathsf {map}: \mathsf {Dom}(\gamma )\rightarrow X$ with the following properties:

1. $$|\mathsf {Dom}(\gamma )| = D^{d}$$.
2. $\gamma _\mathsf {map}$ maps $\mathsf {Dom}(\gamma )$ to the set of simplices contained in the path ${\gamma }$.

We will utilize the following construction given in Berger (1995).

#### Definition 12

Let $\gamma \in IX_{d-1}$ . We define $\mathsf {Dom}(\gamma )$ and $\gamma _\mathsf {map}$ as follows. Suppose, that $\gamma = [y_1,i_1]^{\epsilon _1}[y_2,i_2]^{\epsilon _2}\cdots [y_k,i_k]^{\epsilon _k}$. For every edge $[y_j, i_j]^{\epsilon _j}$, let $\alpha _j$ be the simplicial map ${\varDelta }^{d} \rightarrow y_j$ sending the nondegenerate *d* simplex in ${\varDelta }^{d}$ to $y_j$.

We define $\mathsf {Dom}(\gamma )$ as a quotient of the disjoint union of *k* copies of ${\varDelta }^{d}$:

$$\begin{aligned} \mathsf {Dom}(\gamma ) = \bigsqcup _{i = 1} ^{k} {\varDelta }^{d} /\sim \end{aligned}$$

where each copy of ${\varDelta }^{d}$ corresponds to a domain of a unique $\alpha _j$ and the relation is given by

$$\begin{aligned} (\alpha _j)^{-1} \mathsf {target} \,([y_j,i_j]^{\epsilon _j}) \sim (\alpha _{j+1})^{-1} \mathsf {source} \,([y_{j+1},i_{j+1}]^{\epsilon _{j+1}}) . \end{aligned}$$

The map $\gamma _\mathsf {map}$ is induced by the collection of maps $\alpha _1, \ldots , \alpha _k$:

We recall that simplicial set ${\overline{I}}X$ was defined as the set of “reduced and compressed” paths in *IX*. Similarly, one introduces a reduced and compressed versions of the construction $\mathsf {Dom}$. As a final step we then get

#### Lemma 14

(Section 2.4 in Berger 1995) Let $\gamma \in {\overline{{\varOmega }}}X_{d-1}$ such that $d_i \gamma = 1\in {\overline{{\varOmega }}}X$ for all *i*. Then the map $\gamma _\mathsf {map}: \mathsf {Dom}(\gamma )\rightarrow X$ factorizes through a simplicial set model of the sphere ${\varSigma }^{d}(\gamma )$ as follows:Further, $\pi _{d-1}({\overline{{\varOmega }}}X)\ni [\gamma ] \simeq [\gamma _\mathsf {sph}] \in \pi _{d}(X)$.

We will not give the proof of correctness of Lemma 14 (it can be found in Berger 1995). Instead, in the next section, we only describe the algorithmic construction of $\gamma _\mathsf {sph}: {\varSigma }^{d}(\gamma ) \rightarrow X$ and give a running time estimate.

### Algorithm from Lemma 14

The algorithm accepts an element $\gamma \in {\overline{{\varOmega }}}X_{d-1}$ such that $d_i \gamma = 1\in {\overline{{\varOmega }}}X$ for all *i*, a spherical element. We divide the algorithm into four steps that correspond to the four step factorization in the following diagram:

$$\mathsf {Dom}(\gamma )$$:
We interpret $\gamma$ as an element in *IX* and construct $\gamma _\mathsf {map}: \mathsf {Dom}(\gamma ) \rightarrow X$. This is clearly linear in the size of $\gamma$.
$${\overline{\mathsf {Dom}}}(\gamma )$$:
The algorithm checks, whether an edge $[y,j]^\epsilon$ in $d_{i_1}d_{i_2}\ldots d_{i_\ell } \gamma$, where $0\le {i_1}< {i_2}< \cdots< {i_\ell }<(d-\ell -2)$ is *compressible*, i.e. $y = s_j d_j y$. If this is the case, add a corresponding relation on the preimages: $\alpha ^{-1}(y) \sim s_j d_j \alpha ^{-1}(y)$. Factoring out the relations, we get a map $\gamma _{\mathsf {c}}: {\overline{\mathsf {Dom}}}(\gamma ) \rightarrow X$.
  Although the number of faces we have to go through is exponential in *d*, this is not a problem, since *d* is deemed as a constant in the algorithm and so is $2^d$. Hence the number of operations is again linear in the size of $\gamma$.
$${\overline{{\overline{\mathsf {Dom}}}}}(\gamma )$$:
Let $k<d$. We know that $\overline{d_k \gamma }=1_*$, so after removing all compressible elements from the path $d_k \gamma$, it will contain a sequence of pairs ($[y_i,j_i]^{\epsilon _i}, [y_i, j_i]^{-\epsilon _i})$ such that, after removing all $[y_u, j_u]^{\pm 1}$ for all $u<v$, then $[y_v, j_v]^{\epsilon _v}$ and $[y_v, j_v]^{-\epsilon _v}$ are next to each other.^17^ Each such pair $([y_i,j_i]^{\epsilon _i}, [y_i, j_i]^{-\epsilon _i})$ corresponds to a pair of indices $(l_i,m_i)$ corresponding to the positions of those edges in $d_k \gamma$. These sequences are not unique, but can be easily found in time linear in $\mathrm {length}(\gamma )$. Then we glue $\alpha _{l_i}^{-1}(y_i)$ with $\alpha _{m_i}^{-1}(y_i)$ for all *i*. Performing such identifications for all *k* defines the new simplicial set ${\overline{{\overline{\mathsf {Dom}}}}}(\gamma )$.
$${\varSigma }^{d}(\gamma )$$:
It remains to identify $\alpha ^{-1}(\mathsf {source} \,\gamma )$ and $\alpha ^{-1} (\mathsf {target} \,\gamma )$ with the appropriate degeneracy of the (unique) basepoint. The resulting space $|{\varSigma }^{d}(\gamma )|$ is a *d*-sphere.

## Polynomial-time loop contraction in $F_d$

In this section, we show that simplicial sets $F_i$, $2\le i \le d$ constructed algorithmically in Sect. 4 have polynomial-time contractible loops, thus proving Lemma 4.

Assuming that *X* is a 0-reduced, 1-connected simplicial set with a given algorithm that computes the contraction on loops $c_0: (GX)_0 \rightarrow (GX)_1$, the contraction $c_0$ on $F_2$ is automatically defined, as $F_2 = X$.

The majority of the effort in the rest of this section is concentrated on the description of the contraction $c_0$ on $F_3$, as show that the contraction $F_i, i>3$ can be easily obtained from the contraction on $F_3$.

We remark that the loop contractions, i.e. maps $c_0 :G_0(F_i) \rightarrow G_1(F_i)$ with $d_0 c_0 = {\mathrm{id}}$ and $d_1 c_0 = 1$, are not unique. We only describe one of possible choices and provide an analysis on the overall length of the formulas/running time of the algorithm.

### Notation

We will further use the following shorthand notation: For a 0-reduced simplicial set *X* we will denote the iterated degeneracy $s_0 \cdots s_0 *$ of its unique basepoint $*$ by $*$ and we set $\pi _i = \pi _i(X)$. For any Eilenberg–Maclane space $K(\pi _i, i-1)$, $i\ge 2$, we denote its basepoint and its degeneracies by 0. From the context, it will always be clear which simplicial set we refer to.

### Loop contraction on $F_i$, $i>3$

Suppose we have defined the contraction on the generators of $G_0(F_3)$. i.e. for any $(x,k) \in (X\times _{\tau '}K(\pi _2, 1))_1$ we have

$$\begin{aligned} c_0(\overline{(x,k)}) = \overline{(x_1,k_1)}^{\epsilon _1} \cdots \overline{(x_{n},k_{n})}^{\epsilon _{n}}\qquad (x_j,k_j) \in (F_3)_2, \epsilon _j \in {\mathbb {Z}}, 1\le j\le {n}\end{aligned}$$

such that $d_0 c_0 (\overline{(x,k)}) = \overline{(x,k)}$ and $d_1 c_0 (\overline{(x,k)}) = 1$. In detail, we get the following:

9 $$\begin{aligned} \overline{(x,k)}&= d_0 c_0(\overline{(x,k)}) =\overline{(d_0 x_1,d_0 k_1)}^{\epsilon _1} \cdots \overline{(d_0 x_{n},d_0 k_{n})}^{\epsilon _{n}} \end{aligned}$$

10 $$\begin{aligned} 1&= d_1 c_0(\overline{(x,k)}) =\big (\overline{(d_2 x_1 , \tau '(x_1) d_2 k_1 )}^{-1}\cdot \overline{(d_1 x_1 ,d_1 k_1)}\big )^{\epsilon _1} \cdots \nonumber \\ { }&\quad \big (\overline{(d_2 x_{n}, \tau '(x_{n}) d_2 k_{n})}^{-1}\cdot \overline{(d_1 x_{n},d_1 k_{n})}\big )^{\epsilon _{n}} \end{aligned}$$

We now aim to give a reduction on the generators of $G_0(F_i)$, $i>3$. Simplicial set ${F_i}$ is an iterated twisted product of the form

$$\begin{aligned} \big ((( X\times _{\tau '} K(\pi _2, 1)) \times _{\tau '} K(\pi _3, 2) )\times _{\tau '} \cdots \times _{\tau '} K(\pi _{i-2}, i-3)\big ) \times _{\tau '} K(\pi _{i-1}, i-2) \end{aligned}$$

As simplicial sets $K(\pi _{i-1}, i-2)$ are 1-reduced for $i>3$, we can identify elements of $({F_i})_1$ with vectors $(x,k,0, \ldots ,0)$, where $k\in K(\pi _{2}, 1)_1, x\in X_1$. We further shorthand the series of $i-3$ zeros in the vector with ${\mathbf {0}}$. Hence generators $G_0(F_i)$ are of the form $\overline{(x,k,{\mathbf {0}})}$. The 1-reducedness also implies that $\tau ' (\alpha ) = 0$ whenever $\alpha \in (F_i)_2$, $i>2$.

Finally, we set

$$\begin{aligned}&c_0(\overline{(x,k,{\mathbf {0}})}) = \overline{(x_1, k_1, {\mathbf {0}})}^{\epsilon _1} \cdots \overline{(x_{n}, k_{n}, {\mathbf {0}})}^{ \epsilon _{n}}\\&\quad (x_j, k_j, {\mathbf {0}}) \in (F_i)_2, \epsilon _j \in {\mathbb {Z}}, 1\le j\le {n}\end{aligned}$$

The (almost) freeness of $G_0(F_i)$, the fact that $K(\pi _{i-1}, i-2)$ are 1-reduced for $i>3$ and Eqs. (9), (10) give that $d_0 c_0(\overline{(x,k,{\mathbf {0}})}) = \overline{(x,k,{\mathbf {0}})}$ and $d_1 c_0(\overline{(x,k,{\mathbf {0}})}) = 1$.

Before the definition of contraction on simplicial set $F_3$, we recall some basic facts about the simplicial model of Eilenberg–MacLane spaces we are using.

### Eilenberg–MacLane spaces

As noted in Sect. 3, given a group $\pi$ and an integer $i\ge 0$ an Eilenberg–MacLane space $K(\pi , i)$ is a space satisfying

$$\begin{aligned} \pi _j (K(\pi , i)) = \left\{ \begin{array}{ll} \pi &{} \quad \text{ for } j = i,\\ 0 &{} \quad \text{ else }. \end{array} \right. \end{aligned}$$

In the rest of this section, by $K(\pi , i)$ we will always mean the simplicial model which is defined in May (1992, p. 101)

$$\begin{aligned} K(\pi , i)_q = Z^i ({\varDelta }^{q}; \pi ), \end{aligned}$$

where ${\varDelta }^{q} \in \mathsf {sSet}$ is the standard *q*-simplex and $Z^i$ denotes the cocycles. This means that each *q*-simplex is regarded as a labeling of the *i*-dimensional faces of ${\varDelta }^{q}$ by elements of $\pi$ such that they add up to $0\in \pi$ on the boundary of every $(i+1)$-simplex in ${\varDelta }^{q}$, hence elements of $K(\pi , q)_q$ are in bijection with elements of $\pi$. The boundary and degeneracy operators in $K(\pi , {k})$ are given as follows: For any $\sigma \in K(\pi , i)_q$, $d_j(\sigma ) \in K(\pi , {k})_{q-1}$ is given by a restriction of $\sigma \in K(\pi , i)$ to the *j*-th face of ${\varDelta }^{q}$. To define the degeneracy we first introduce mapping $\eta _j :\{0,1, \ldots , q+1 \} \rightarrow \{0,1, \ldots , q\}$ given by

$$\begin{aligned} \eta _j (\ell )= \left\{ \begin{array}{ll} \ell &{} \quad \text{ for } \ell \le j, \\ \ell -1 &{} \quad \text{ for } \ell > j. \end{array} \right. \end{aligned}$$

Every mapping $\eta _j$ defines a map $C^*(\eta _j ):C^*({\varDelta }^{q}) \rightarrow C^*({\varDelta }^{q+1})$.The degeneracy $s_j \sigma$ is now defined to be $C^*(\eta _j)(\sigma )$ (see May 1992, §23).

It follows from our model of Eilenberg–MacLane space, that elements of $K(\pi _2, 1)_2$ can be identified with labelings of 1-faces of a 2-simplex by elements of $\pi _2$ that sum up to zero.

As $\pi _2$ is an Abelian group, we use the additive notation for $\pi _2$. We identify the elements of $K(\pi _2, 1)_2$ with triples $(k_0, k_1, k_2)$, $k_i \in \pi _2$, $0\le i\le 2$, such that $k_0 - k_1 + k_2 = 0 \in \pi _2$.

### Loop contraction on $F_3$

Let *X* be a 0-reduced, 1-connected simplicial set with a given algorithm that computes the contraction on loops $c_0: (GX)_0 \rightarrow (GX)_1$.

In the rest of the section, we will assume $x \in X_1$. Then by our assumptions $c_0 {\overline{x}} = \overline{y_1}^{\epsilon _1} \cdots \overline{y_n}^{\epsilon _n}$, where $y_i \in X_2, \epsilon _i \in {\mathbb {Z}}$, $1\le i\le n$. Let $k_i = \tau ' (y_i)$.

We first show that in order to give a contraction on elements of the form $\overline{(x,0)}$ and $\overline{(x,k)}$, it suffices to have the contraction on elements of the form $\overline{(*,k)}$:

### Contraction on element (*x*, 0)

Let $\overline{(x,0)} \in G_0 (F_3)$. We define

$$\begin{aligned} c_0 \overline{(x,0)} = \prod _{i = 1} ^{n}\big ( c_0 \overline{(*,k_i)}^{-1} \overline{( s_1 d_2 y_i,(k_i, k_i, 0))}\cdot \overline{(y_i,0)}\big )^{\epsilon _i}. \end{aligned}$$

### Contraction on element (*x*, *k*)

Suppose $\overline{(x,k)} \in (GF_3)_0$. The formula for the contraction is given using the formulae on contraction on $\overline{(x,0)}$ and $\overline{(*,k)}$ as follows

$$\begin{aligned} c_0 \overline{(x,k)} = \overline{(s_0 x,(k, 0,-k))} \cdot s_0 \overline{(x, 0)}^{-1} \cdot s_0 \overline{(*, -k)} \cdot c_0 (\overline{(*,-k)})^{-1}\cdot c_0 (\overline{(x,0)}) \end{aligned}$$

### Contraction on element $(*,k)$

We formalize the existence of the contraction as follows:

#### Lemma 15

Let $k\in \pi _2(X)$. Then there is an algorithm that computes an element $z\in (GF_3)_1$ such that $d_0 z = \overline{(*,k)}$ and $d_1 z = 1$.

The proof is postponed until later and uses a variety of technical results. The main idea is to take a generator of $\pi _2(X)$ represented by a spherical element $\gamma \in \pi _1(GX)$ with $[\gamma ] = k$, and use it to find a filler of $\overline{(*,k)}$.

Consider first the following, simplified, situation: Suppose that there is $y\in (X)_2$ with $d_0(\overline{y}) = 1$ and $d_1(\overline{y}) = \overline{d_2 y}^{-1} \overline{d_1 y} = 1$. Let $k = [\overline{y}]\in \pi _2(X)$, then $\tau '(y) = k$. Observe that

$$\begin{aligned} d_0 \overline{(y,(0,0,0))}&=\overline{(d_0 y, 0)} = \overline{(*, 0)} = 1 \\ d_1 \overline{(y,(0, 0, 0))}&=\overline{(d_2 y, k )}^{-1}\cdot \overline{(d_1 y ,0)}. \end{aligned}$$

Purely from the point of view of information at our disposal, we are close to finding $c_0\overline{(*,k)}$. What we need to do is to employ some algebraic machinations that would “uncouple” the pair $\overline{(d_2 y, k )}^{-1}$ into, say $\overline{(d_2 y, 0 )}^{-1}$ and $\overline{(*, k )}^{-1}$, “merge” $\overline{(d_2 y, 0 )}^{-1}$ with $\overline{(d_1 y, 0 )}$ (thus eliminating it) and , finally, “switch” the “$d_0$” and “$d_1$”. These informally described operations are made precise in Lemma 17.

In general case, for an arbitrary $k\in \pi _2$ one cannot expect that there exists *y* with the properties as above, however, the following is true:

#### Lemma 16

Let $k\in \pi _2$, then there exists $\gamma = \overline{y_1}^{\epsilon _1}\cdots \overline{y_n}^{\epsilon _n} \in {\widetilde{GX}}_1$ where $y_i \in X_2$ and $\tau ' y_i = k_i \in \pi _2 (X)$, such that $d_0 \gamma = 1 = d_1 \gamma$ and $\sum _{i=1}^{n} \epsilon _i \cdot k_i = k$.

#### Proof

Given an element $k\in \pi _2 \cong H_2(X)$, one can compute a cycle $\gamma ' \in Z_2 (X)$ such that

$$\begin{aligned}{}[\gamma '] = k \in \pi _2(X) \cong H_2(X) \cong H_2(K(\pi _2, 2)) \cong \pi _2(K(\pi _2, 2)), \end{aligned}$$

where the middle isomorphism is induced by $\varphi _2$ and the other isomorphisms follow from the Hurewicz theorem.

We recall that the loop contraction $c_0$ on the space *X* is given as a part of the input. According to Lemma 13, one can compute the inverse of the Hurewicz isomorphism

$$\begin{aligned} H_{1}({\widetilde{GX}})\rightarrow H_{1}({\widetilde{AX}})\cong H_2(X) \end{aligned}$$

on the level of representatives.

Therefore, if we consider $\gamma ' \in {\widetilde{AX}}_1$, we can algorithmically compute a spherical element $\gamma = \overline{y_1}^{\epsilon _1}\cdots \overline{y_n}^{\epsilon _n} \in {\widetilde{GX}}_1$ and it is easy to see that it has the desired properties. $\square$

We can now use Lemma 16 and some minor technical tricks to prove Lemma 15.

#### Proof of Lemma 15

Let $k\in \pi _2$, then, by Lemma 16, we compute $\gamma = \overline{y_1}^{\epsilon _1}\cdots \overline{y_n}^{\epsilon _n} \in {\widetilde{GX}}_1$ with $y_i \in X_2$ and $\tau ' y_i = k_i \in \pi _2 (X)$, such that $d_0 \gamma = 1 = d_1 \gamma$ and $\sum _{i=1}^{n} \epsilon _i \cdot k_i = k$.

We define $z' \in (GF_3)_1$ by

$$\begin{aligned} z' = \left( \prod _{i = 1} ^{n} \overline{(s_0 d_0 y_i,(k_i, 0, -k_i))}^{\epsilon _i}\right) \cdot \left( \prod _{i = 1} ^{n} \overline{(y_i,(k_i, 0, -k_i))}^{\epsilon _i}\right) ^{-1}. \end{aligned}$$

Observe that $d_0(z') = 1$ and

$$\begin{aligned} d_1 z' = \big (\overline{(*, -k_1)}^{-1} \cdot \overline{(d_0 y_1,0 )}\big )^{\epsilon _1}\cdots \big (\overline{(*, -k_n)}^{-1} \cdot \overline{(d_0 y_n,0)}\big )^{\epsilon _n}. \end{aligned}$$

We apply Lemma 18 on $z'$ and get an element $z'' \in (GF_3)_1$ with the property $d_0 z'' = 1$ and $d_1 z'' = \overline{(*, k)}$. We define $z = s_0 \overline{(*, k)} \cdot (z'')^{-1}$. Thus $d_0 z = \overline{(*,k)}$ and $d_1 z = 1$.$\square$

### Technical statements

#### Definition 13

Let $Z = \{z \in (GF_3)_1 \mid d_0 z = 1\}$ and let $W = \{d_1 z \mid z\in Z \}$ We define an equivalence relation $\sim$ on the elements of *W* in the following way: We say that $w\sim w'$ if there exists $z \in Z$, $\alpha , \beta \in (GF_3)_1$ such that $d_1 z = w$, $\alpha z \beta \in Z$ and $d_1(\alpha z \beta ) = w'$.

#### Lemma 17

Let $w\in W$ such that

1. $$w= \overline{(x,k)}^{\epsilon }\cdot \alpha $$, where $$\alpha \in (GF_3)_1$$ Then $$w = \overline{(x,k)}^{\epsilon }\cdot \alpha \sim \alpha \cdot (x,k)^{\epsilon } = w'$$.
2. $$w =\overline{(*,k)}^{\epsilon } \cdot \alpha $$, where $$\alpha \in (GF_3)_0$$. Then $$w \sim w'= \overline{(*,-k)}^{-\epsilon } \cdot \alpha $$.
3. $$w = \overline{(*,- k)}^{-1} (x,0)\cdot \alpha $$, where $$\alpha \in (GF_3)_0$$. Then $$w \sim w' = \overline{(x,k)}\cdot \alpha $$.
4. $$w = \overline{(x,0)}^{-1} \overline{(x,k)} \cdot \alpha $$, where $$\alpha \in (GF_3)_0$$. Then $$w \sim w'= \overline{(*,k)} \cdot \alpha $$.
5. $$w = \overline{(*, -l)}^{-1} \overline{(*,k)} \cdot \alpha $$, where $$\alpha \in (GF_3)_0$$. Then $$w \sim w'= \overline{(*,k+l)}\cdot \alpha $$.

#### Proof

In all cases, we assume $z\in Z$ such that $d_1 z = w$ and we give a formula for $z' \in Z$ with $d_1 z' = w'$:

1. $$z' = s_0 \overline{(x.k)}^{-\epsilon } \cdot z \cdot s_0 \overline{(x,k)}^{\epsilon }$$.
2. $$z' = \overline{(*,(k, 0, -k))}^{\epsilon } \cdot (s_0\overline{(*,k)})^{-\epsilon } \cdot z$$.
3. $$z' = (s_0\overline{(x,k)}) \cdot \overline{(s_0 x,(k, 0, -k))}^{-1} \cdot z$$.
4. $$z' = (s_0\overline{(*,k)})\overline{(s_1 x,(k,k,0))}^{-1}\cdot z$$.
5. $$z' = \overline{(s_0(*,k + l))}\overline{(*,(k + l,k, - l))}^{-1}\cdot z$$.

$$\square $$

#### Lemma 18

Let $$z \in (GF_3)_1$$, $$z\in Z$$ with

$$\begin{aligned} d_1 z = w = \overline{(*,-k_1)}^{-1}\cdot \overline{(x_1, 0)}^{\epsilon _1} \cdots \overline{(*,-k_n)}^{-1} \cdot \overline{(x_n, 0)}^{\epsilon _n} \end{aligned}$$

where $\overline{x_1}^{\epsilon _1} \cdots \overline{x_n}^{\epsilon _n} = 1$ in $GX_0$, $x_i \in X$, $k_i \in \pi _2(X)$, $\epsilon _i \in \{1,-1\}$, $1\le i\le n$. Then $w \sim \overline{(\sum _{i = 1} ^{n} k_i, *)}$.

#### Proof

We achieve the proof using a sequence of equivalences given in Lemma 17. Without loss of generality we can assume that $x_1 = x_2^{-1}$ and $\epsilon _1, \epsilon _2 = 1$ (If this is not the case, we can use rule (1) and/or relabel the elements). Using (1) gives us

$$\begin{aligned} w&= \overline{(*,-k_1)}^{-1} \cdot \overline{(x_2,0)}^{-1} \cdot \overline{(*, -k_2)}^{-1} \cdot \overline{(x_2,0)} \cdots \overline{(*,-k_n)}^{-1} \cdot \overline{(x_n,0)}^{\epsilon _n} \\&\quad \sim \overline{(*, -k_2)}^{-1} \cdot \overline{(x_2,0)}\cdots \overline{(*,-k_n)}^{-1} \cdot \overline{(x_n,0)}^{\epsilon _n}\cdot \overline{(*,-k_1)}^{-1} \cdot \overline{(x_2,0)}^{-1}. \end{aligned}$$

Then successive use of (3), (1), (4), (1) and finally (5) gives us

$$\begin{aligned} w&\sim \overline{(x_2,k_2)}\cdots \overline{(*,-k_n)}^{-1} \cdot \overline{(x_n,0)}^{\epsilon _n}\cdot \overline{(*,-k_1)}^{-1} \cdot \overline{(x_2,0)}^{-1}.\\&\sim \overline{(x_2,0)}^{-1}\cdot \overline{(x_2,k_2)}\cdots \overline{(*,-k_n)}^{-1} \cdot \overline{(x_n,0)}^{\epsilon _n}\cdot \overline{(*,-k_1)}^{-1} \\&\sim \overline{(*,k_2)}\cdots \overline{(*,-k_n)}^{-1} \cdot \overline{(x_n,0)}^{\epsilon _n}\cdot \overline{(*,-k_1)}^{-1}\\&\sim \overline{(*,k_1 +k_2)}\cdot \overline{(*,-k_3)}^{-1} \cdot \overline{(x_3, 0)}\cdots \overline{(*,-k_n)}^{-1} \cdot \overline{(x_n,0)}^{\epsilon _n} \end{aligned}$$

multiple use or rules (2) and (1) and gives us

$$\begin{aligned} w \sim&\overline{(*, - k_1 - k_2 - k_3)}^{-1} \cdot \overline{(x_3, 0)}\cdots \overline{(*,-k_n)}^{-1} \cdot \overline{(x_n,0)}^{\epsilon _n} \end{aligned}$$

So far, we have produced some element $z' \in Z \subseteq (GF_3)_1$ such that $d_0 z' = 1$,

$$\begin{aligned} d_1 z' = \overline{(*, - k_1 - k_2 - k_3)}^{-1} \cdot \overline{(x_3, 0)}\cdots \overline{(*,-k_n)}^{-1} \cdot \overline{(x_n,0)}^{\epsilon _n} \end{aligned}$$

and further $\overline{x_3}^{\epsilon _3} \cdots \overline{x_n}^{\epsilon _n}= 1$ in $GX_0$.

It follows that the construction described above can be applied iteratively until all elements $\overline{(x_i, 0)}$ are removed and we obtain $w \sim \overline{( -\sum _{i = 1} ^{n} k_i, *)}^{-1} \sim \overline{(\sum _{i = 1} ^{n} k_i, *)}$. $\square$

### Computational complexity

We first observe that formulas for $c_0$ on a general element $\overline{(x,k)}$ depend polynomially on the size of $c_0 (\overline{x})$ and the size of contractions on $\overline{(*,k)}$. Hence it is enough to analyze the complexity of the algorithm described in Proposition 15.

The computation of $\gamma '$ is obtained by the polynomial-time Smith normal form algorithm presented in Kannan and Bachem (1981) and the polynomial-time algorithm in Lemma 13. The size of $z'$ depends polynomially (in fact linearly) on size of $\gamma '$. The algorithm described in Lemma 18 runs in a linear time in the size of $z'$.

To sum up, the algorithm computes the formula for contraction on the elements of $GF_i$ in time polynomial in the input ($\mathrm {size}\, X + \mathrm {size}\, c_0 (GX)$).

## Reconstructing a map to the original simplicial complex

This section contains the proof of Lemma 6 formulated on page 20. To summarize it, we will prove that whenever a simplicial set *X* was constructed from a simplicial complex $X^{sc}$ by contracting its spanning tree *T* into the basepoint and ${\varSigma }$ is a simplicial complex, then we can convert a simplicial map ${\varSigma }\rightarrow X$ into a simplicial map between *simplicial complexes*$Sd({\varSigma })\rightarrow X^{sc}$ where $Sd({\varSigma })$ is a suitable subdivision of ${\varSigma }$. We will describe an explicit construction and argue that the underlying algorithm is polynomial.

**Fig. 4:**
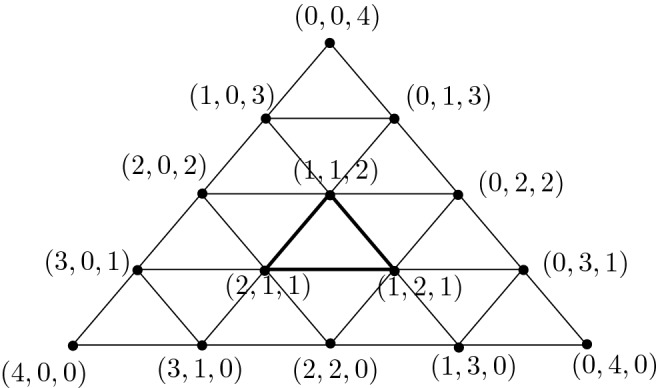
Edgewise subdivision of a 2-simplex for $k=4$. In this case, there exists a copy of the 2-simplex completely in the “interior”, defined by vertices (2, 1, 1), (1, 2, 1) and (1, 1, 2). All other vertices are at the “boundary”: more formally, their coordinates contain a zero

### Edgewise subdivision of simplicial complexes

In Edelsbrunner and Grayson (1999), the authors present, for $k\in {\mathbb {N}}$, the *edgewise subdivision*$\mathrm {Esd}_k({\varDelta }^m)$ of an *m*-simplex ${\varDelta }^m$ that generalizes the two-dimensional sketch displayed in Fig. 4. This subdivision has several nice properties: in particular, the number of simplices of $\mathrm {Esd}_k({\varDelta }^m)$ grows polynomially with *k*. Explicitly, the subdivision can be represented as follows.

- The vertices of $\mathrm {Esd}_k({\varDelta }^m)$ are labeled by integer coordinates $(a_0,\ldots , a_{m})$ such that $a_j\ge 0$ and $\sum _j a_j=k$.
- Two vertices $(a_0,\ldots a_m)$ and $(b_0,\ldots , b_m)$ are *adjacent* if there is a pair $j<k$ such that $|b_j-a_j|=|b_k-a_k|=1$ and $a_i=b_i$ for $i\ne j,k$.
- Simplices of $\mathrm {Esd}_k({\varDelta }^m)$ are given by tuples of vertices such that each vertex of a simplex is adjacent to each other vertex.

We define the *distance* of two vertices to be the minimal number of edges between them.

An edgewise *k*-subdivision of ${\varDelta }^m$ induces an edgewise *k*-subdivision of all faces, hence we may naturally define an edgewise subdivision of any simplicial complex.

### Constructing the map $\mathrm {Esd}_k({\varSigma })\rightarrow X^{sc}$

Let *R* be a chosen root in the tree *T*. We denote the tree-distance of a vertex *W* from *R* by $\mathrm {dist}_T(W)$. Let

$$\begin{aligned} l:=\max \{\mathrm {dist}_T(V):\,\,V\,\text { is a vertex of } X^{sc}\} \end{aligned}$$

be the maximal tree-distance of some vertex from *R*. For each vertex *V* of $X^{sc}$, there is a unique path in the spanning tree that goes from *R* into *V*. Further, we define the maps $M(j): (X^{sc})^{(0)}\rightarrow (X^{sc})^{(0)}$ from vertices of $X^{sc}$ into vertices of $X^{sc}$ such that

- $$M(j)(V):=V$$ if $$j\ge \mathrm {dist}_T(V)$$, and
- *M*(*j*)(*V*) is the vertex on the unique tree-path from *R* to *V* that has tree-distance *j* from *R* if $j < \mathrm {dist}_T(V)$.

If, for example, $R-U-V-W$ is a path in the tree, then $M(0)(W)=R$, $M(1)(W)=U$ etc. Clearly, $M(l)=M(l+1)=\cdots$ is the identity map, as *l* equals the longest possible tree-distance of some vertex.

Assume that *d* is the dimension of ${\varSigma }$ and $k:=l(d+1)+1$. We will define $f': \mathrm {Esd}_k({\varSigma })\rightarrow X^{sc}$ simplexwise. Let $\tau \in {\varSigma }$ be an *m*-simplex and $f(\tau )={\tilde{\sigma }}\in {X}$ be its image in the simplicial set *X*. If ${\sigma }$ is the degeneracy of the base-point $*\in X$, then we define $f'(x):=R$ for all vertices *x* of $\mathrm {Esd}_k(\tau )$: in other words, $f'$ will be constant on the subdivision of $\tau$. Otherwise, ${\tilde{\sigma }}$ is not the degeneracy of a point and has a unique lift $\sigma \in X^{ss}$ (recall that $X:=X^{ss}/T$). Let $(V_0,\ldots , V_m)$ be the vertices of $\sigma$ (order given by orientation): these vertices are not necessarily different, as $\sigma$ may be degenerate.

In the algorithm, we will need to know which faces of $\sigma$ are in the tree *T*. We formalize this as follows: let $S\subseteq 2^m$ be the family of all subsets of $\{0,1,\ldots , m\}$ such that

- For each $\{i_0,\ldots , i_j\}\in S$, $\{V_{i_0}, \ldots , V_{i_j}\}$ is in the tree (that is, it is either an edge or a single vertex),
- Each set in *S* is maximal wrt. inclusion.

Elements of *S* correspond to maximal faces of $\sigma$ that are in the tree, in other words, to faces of ${\tilde{\sigma }}$ that are degeneracies of the base-point.

#### Definition 14

Let ${\varDelta }^m$ be an oriented *m*-simplex, represented as a sequence of vertices $(e_0,\ldots , e_m)$. For any face $s\subseteq \{e_0,\ldots , e_m\}$, we define the *extended face*${\mathcal {E}}(s)$*in*$\mathrm {Esd}_k({\varDelta }^m)$ to be the set of vertices $(x_0,\ldots , x_m)$ in $\mathrm {Esd}_k({\varDelta }^m)$ that have nonzero coordinates only on positions *i* such that $e_i\in S$.

**Fig. 5:**
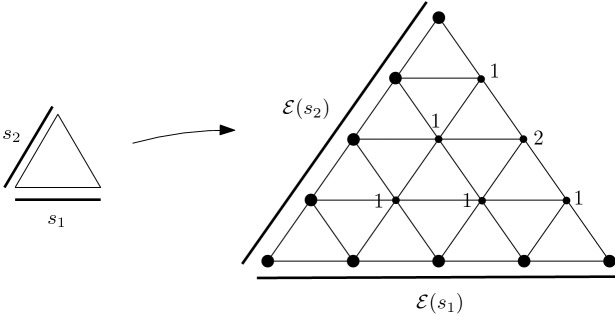
Illustration of extended faces. Here $S=\{s_1,s_2\}$ corresponds to the lower- and left-face of a 2-simplex. The extended faces ${\mathcal {E}}(s_1)$ and ${\mathcal {E}}(s_2)$ are sets of vertices of $\mathrm {Esd}_k({\varDelta }^2)$ that are on the lower- and left-boundary. The corresponding extended tree ${\mathcal {E}}(T)$ is the union of all these vertices. The integers indicate edge-distances ${{\mathrm{dist}}}_{ET}$ of vertices in $\mathrm {Esd}_k({\varDelta }^2)$ from ${\mathcal {E}}(T)$

The geometric meaning of this is illustrated by Fig. 5.

#### Definition 15

Let *S* be defined as above. We define the *extended tree*${\mathcal {E}}(T)$ to be the union of the extended faces ${\mathcal {E}}(s)$ in $\mathrm {Esd}_k({\varDelta }^m)$ for all $s\in S$. The edge-distance of a vertex *x* in $\mathrm {Esd}_k({\varDelta }^m)$ from ${\mathcal {E}}(T)$ will be denoted by $\mathrm {dist}_{ET}(x)$.

In words, ${\mathcal {E}}(T)$ it is the union of all vertices in parts of the boundary of $\mathrm {Esd}_k({\varDelta }^m)$ that correspond to the faces of $\sigma$ that are in the tree, see Fig. 5. The number $\mathrm {dist}_{ET}(x)$ is the distance to *x* from those boundary parts that correspond to faces of $\sigma$ that are in the tree.

**Fig. 6:**
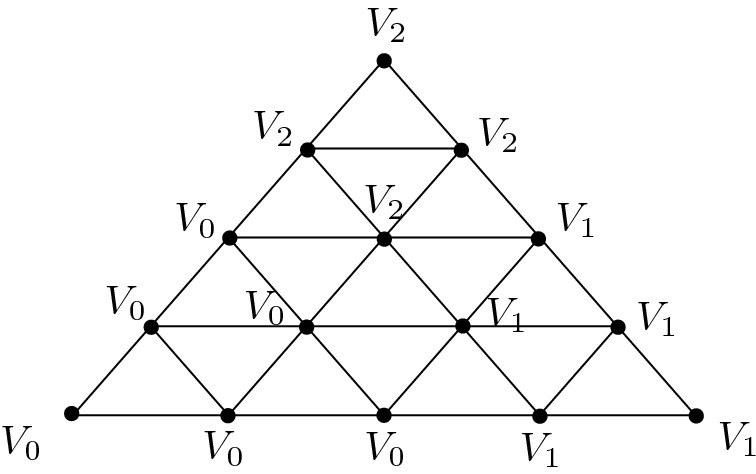
Labeling vertices of $\mathrm {Esd}_k({\varDelta }^2)$ by $V_{{{\mathrm{arg\,max}}}x}$

To define a simplicial map from $\mathrm {Esd}_k(\tau )$ to $X^{sc}$, we need to label vertices of $\mathrm {Esd}_k(\tau )$ by vertices of $X^{sc}$ such that the induced map takes simplices in $\mathrm {Esd}_k(\tau )$ to simplices in $X^{sc}$. Recall that $V_0,\ldots ,V_m$ are the vertices of $\sigma$. For $x=(x_0,\ldots , x_m)$, we denote by ${{\mathrm{arg\,max}}}\,x$ the smallest index of a coordinate of *x* among those with maximal value [for instance, ${{\mathrm{arg\,max}}}\,(4,2,1,4,0)=0$, as the first 4 is on position 0]. The geometric meaning of $V_{{{\mathrm{arg\,max}}}x}$ is illustrated by Fig. 6.

Now we are ready to define the map $f': \mathrm {Esd}_k(\tau )\rightarrow X^{sc}$. It is defined on vertices *x* with coordinates $(x_0,\ldots ,x_m)$ by

11 $$\begin{aligned} f'(x_0,\ldots , x_m) := M(\mathrm {dist}_{ET}(x))(V_{{{\mathrm{arg\,max}}}\,\,x}). \end{aligned}$$

Geometrically, most vertices *x* will be simply mapped to $V_j$ for which the *j*’th coordinate of *x* is dominant. In particular, a unique *m*-simplex “most in the interior of $\mathrm {Esd}_k(\tau )$” with coordinates

12 $$\begin{aligned} \begin{pmatrix} j+1\\ j\\ \ldots \\ j \\ \hline j+1 \\ \ldots \\ j+1 \end{pmatrix}^T, \begin{pmatrix} j\\ j+1\\ \ldots \\ j \\ \hline j+1 \\ \ldots \\ j+1 \end{pmatrix}^T, \ldots , \begin{pmatrix} j\\ j\\ \ldots \\ j+1 \\ \hline j+1 \\ \ldots \\ j+1 \end{pmatrix}^T, \begin{pmatrix} j\\ j\\ \ldots \\ j \\ \hline j+2 \\ \ldots \\ j+1 \end{pmatrix}^T, \ldots , \begin{pmatrix} j\\ j\\ \ldots \\ j \\ \hline j+1 \\ \ldots \\ j+2 \end{pmatrix}^T \end{aligned}$$

for suitable *j* will be labeled by $V_0,V_1,\ldots , V_m$; in other words, it will be mapped to $\sigma$.^18^

**Fig. 7:**
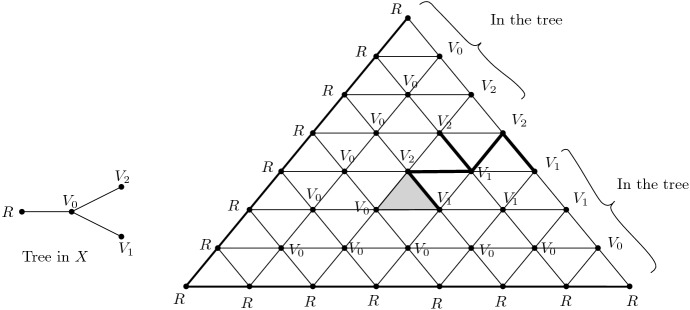
Example of the labeling induced by formula (11). We assume that $f(\tau )={\tilde{\sigma }}$ where $\sigma$ is a simplex of $X^{sc}$ with three different vertices $V_0 V_1 V_2$. In this example, the tree connects $R-V_0-V_1$ as well as $R-V_0-V_2$ and the edge $V_1V_2$ is *not* in the tree. On the right, we give the induced labeling of vertices of $\mathrm {Esd}_k(\tau )$ which determines a simplicial map to $X^{sc}$. The bottom and left faces of $\sigma$ are in the tree, hence the bottom and left extended faces in $\mathrm {Esd}_k(\tau )$ are all mapped into *R*. The right face of $\sigma$ is the edge $V_1 V_2$ that is not in the tree: the corresponding right extended face in $\mathrm {Esd}_k(\tau )$ is mapped to a loop $R-V_0-V_1-V_2-V_0-R$, where $V_1 V_2$ is the only part that is *not* in the tree. The bold edges are sent to the edge $V_1 V_2$. The most interior simplex in $\mathrm {Esd}_k(\tau )$ is highlighted and is the only one mapped to $\sigma$

However, vertices *x* close to those boundary parts of $\mathrm {Esd}_k(\tau )$ that correspond to the tree-parts of $\sigma$, will be mapped closer to the root *R* and all the extended tree ${\mathcal {E}}(T)$ will be mapped to *R*. One illustration is in Fig. 7.

### Computational complexity

Assuming that we have a given encoding of ${\varSigma },f,{X},X^{sc}$ and a choice of *T* and *R*, defining a simplicial map $f':\mathrm {Esd}_k({\varSigma })\rightarrow X^{sc}$ is equivalent to labeling vertices of $\mathrm {Esd}_k({\varSigma })$ by vertices of $X^{sc}$. Clearly, the maximal tree-distance *l* of some vertex depends only polynomially on the size of $X^{sc}$ and can be computed in polynomial time, as well as the maps $M(0),\ldots , M(l)$. Whenever $j>l$, we can use the formula $M(j)=\mathrm {id}$. Further, $k=l(d+1)+1$ is linear in *l*, assuming the dimension *d* is fixed. If $\tau \in {\varSigma }$ is an *m*-simplex, then the number of vertices in $\mathrm {Esd}_k(\tau )$ is polynomial^19^ in *k*, and their coordinates can be computed in polynomial time. Finding the lift $\sigma$ of $f(\tau )={\tilde{\sigma }}$ is at most a linear operation in $\mathrm {size}(X^{sc})+\mathrm {size}({\tilde{\sigma }})$. Converting $\sigma \in X^{ss}$ into an ordered sequence $(V_0,V_1,\ldots , V_m)$ amounts to computing its vertices $d_0 d_1 \ldots \hat{d_i}\ldots , d_m \sigma$, where $d_i$ is omitted. Collecting information on faces of $\sigma$ that are in the tree and the set of vertices ${\mathcal {E}}(T)$ is straight-forward: note that assuming fixed dimensions, there are only constantly many faces of each simplex to be checked. If $s=\{i_0,\ldots , i_j\}$ is a face, then the edge-distance of a vertex *x* from ${\mathcal {E}}(s)$ equals to $\sum _{u} x_{i_u}$. Applying formula (11) to *x* requires to compute the edge-distance of *x* from ${\mathcal {E}}(T)$: this equals to the minimum of the edge-distances of *x* from ${\mathcal {E}}(s)$ for all faces *s* of $\sigma$ that are in the tree. Computing ${{\mathrm{arg\,max}}}x$ is a trivial operation. Finally, the number of simplices $\tau$ of ${\varSigma }$ is bounded by the size of ${\varSigma }$, so applying (11) to each vertex of $\mathrm {Esd}_k({\varSigma })$ only requires polynomially many steps in $\mathrm {size}({\varSigma },f,X^{sc},T,{X})$.

### Correctness

What remains is to prove that formula (11) defines a well-defined simplicial map and that $|\mathrm {Esd}_k({\varSigma })|\rightarrow |X^{sc}|\rightarrow |{X}|$ is homotopic to $|{\varSigma }|\rightarrow |{X}|$.

#### Lemma 19

The above algorithm determines a well-defined simplicial map $\mathrm {Esd}({\varSigma })\rightarrow X^{sc}$.

#### Proof

First we claim that formula (11) defines a global labeling of vertices of $\mathrm {Esd}_k({\varSigma })$ by vertices of $X^{sc}$. For this we need to check that if $\tau '$ is a face of $\tau$, then (11) maps vertices of $\mathrm {Esd}_k(\tau ')$ compatibly. This follows from the following facts, each of them easily checkable:

- If $\tau '$ is spanned by vertices of $\tau$ corresponding to $s\subseteq \{0,\ldots , m\}$, then a vertex $x':=(x_0,\ldots ,x_j)$ in $\mathrm {Esd}_k(\tau ')$ has coordinates *x* in $\mathrm {Esd}_k(\tau )$ equal to zero on positions $\{0\ldots , m\}{\setminus } s$ and to $x_0,\ldots , x_j$ on other positions, successively.
- If $V_k':=V_{i_k}$ for $s=(i_0,\ldots ,i_j)$ are the vertices of the corresponding face of $\sigma$, then
  $$\begin{aligned} V_{{{\mathrm{arg\,max}}}x'}'=V_{{{\mathrm{arg\,max}}}x} \end{aligned}$$
- The extended tree $\mathcal {E'}(T)$ in $\mathrm {Esd}_k(\tau ')$ equals the intersection of the extended tree in $\mathrm {Esd}_k(\tau )$ with ${\mathcal {E}}(\tau ')$
- The distance $\mathrm {dist}_{ET}(x')$ in $\mathrm {Esd}_k(\tau ')$ equals $\mathrm {dist}_{ET}(x)$ in $\mathrm {Esd}_k(\tau )$.

Further, we need to show that this labeling defines a well-defined simplicial map, that is, it maps simplices to simplices. We claim that each simplex in $\mathrm {Esd}_k(\tau )$ is mapped either to some subset of $\{V_0,\ldots , V_m\}$ or to some edge in the tree *T*, or to a single vertex.

We will show the last claim by contradiction. Assume that some simplex is *not* mapped to a subset of $\{V_0,\ldots , V_m\}$, and also it is *not* mapped to an edge of the tree and *not* mapped to a single vertex. Then there exist two vertices *x* and *y* in this simplex that are labeled by *U* and *W* in $X^{sc}$, such that either *U* or *W* is not in $\{V_0,\ldots , V_m\}$, *UW* is not in the tree, and $U\ne W$.

The fact that at least one of $\{U,W\}$ does not belong to $\{V_0\ldots , V_m\}$, implies that ${{\mathrm{dist}}}_{ET}(x)<l$ or ${{\mathrm{dist}}}_{ET}(y)<l$ (as *M*(*j*) maps each $V_{{{\mathrm{arg\,max}}}x}$ to itself for $j\ge l$).

Without loss of generality, assume that ${{\mathrm{arg\,max}}}x=0$ and ${{\mathrm{arg\,max}}}y=1$. Then the coordinates of *x* and *y* are either

$$\begin{aligned} x=(j+1,j,x_3,\ldots , x_m),\quad y=(j,j+1,x_3,\ldots , x_m) \end{aligned}$$

such that $x_i\le j+1$ for all $i\ge 3$, or

$$\begin{aligned} x=(j,j,x_3,\ldots , x_m),\quad y=(j-1,j+1,x_3,\ldots , x_m) \end{aligned}$$

for some *j* such that $x_i\le j$ for all $i\ge 3$.

We claim that $V_0\ne V_1$ and that the edge $V_0V_1$ is *not* in the tree. This is because there exists a tree-path from *R* via *U* to $V_0$ and also a tree-path from *R* via *W* to $V_1$ (and $U\ne W$): both $V_0=V_1$ as well as a tree-edge $V_0 V_1$ would create a circle in the tree. In coordinates, this means that vertices $(*,*,0,0,\ldots , 0)$ are not contained in ${\mathcal {E}}(T)$, apart of $(k,0,0,\ldots , 0)$ and $(0,k,0,\ldots , 0)$. So, any vertex in ${\mathcal {E}}(T)$ has a zero on either the zeroth or the first coordinate. This immediately implies that ${{\mathrm{dist}}}_{ET}(x)\ge j$ and ${{\mathrm{dist}}}_{ET}(y)\ge j$. Keeping in mind that coordinates of *x* (and *y*) has to sum up to $k=l(d+1)+1$, the smallest possible value of *j* is $j=l$ (if $m=d$ is maximal), in which case $x=(l+1, l,l,\ldots ,l)$ and $y=(l,l+1,\ldots , l)$. This choice, however, would contradict the fact that either ${{\mathrm{dist}}}_{ET}(x)<l$ or ${{\mathrm{dist}}}_{ET}(y)<l$. Therefore we have a strict inequality $j>l.$ Finally, we derive a contradiction having either ${{\mathrm{dist}}}_{ET}(x)\ge j>l>{{\mathrm{dist}}}_{ET}(x)$, or a similar inequality for *y*.

This completes the proof that each simplex is either mapped to a subset of $\{V_0,\ldots , V_m\}$ or to an edge in the tree or to a single vertex: the image is a simplex in $X^{sc}$ in either case. $\square$

#### Lemma 20

The geometric realizations of $pf': \mathrm {Esd}_k({\varSigma })\rightarrow {X}$ and $f: {\varSigma }\rightarrow {X}$ are homotopic.

#### Proof

First we reduce the general case to the case when all maximal simplices in ${\varSigma }$ (wrt. inclusion) have the same dimension *d*. If this were not the case, we could enrich any lower-dimensional maximal simplex $\tau =\{x_0,\ldots , x_j\}\in {\varSigma }$ by new vertices $y_{j+1}^\tau ,\ldots , y_d^\tau$ and produce a maximal *d*-simplex

$$\begin{aligned} {\tilde{\tau }}=\{x_0,\ldots , x_j, y_{j+1}^\tau ,\ldots , y_d^\tau \}. \end{aligned}$$

Thus we produce a simplicial complex ${\tilde{{\varSigma }}}\supseteq {\varSigma }$ with the required property. Whenever $f(\tau )$ is mapped to ${\tilde{\sigma }}$ where $\sigma =(V_0,\ldots ,V_j)$, we define $f({\tilde{\tau }})$ to be $s_j^{d-j}{\tilde{\sigma }}$, a degenerate simplex with lift $(V_0,\ldots ,V_j,V_j,\ldots , V_j)$. The map $f': {\tilde{{\varSigma }}}\rightarrow X^{sc}$ is constructed from $f: {\tilde{{\varSigma }}}\rightarrow X$ as above and if we prove that |*f*| is homotopic to $|pf'|$ as maps $|{\tilde{{\varSigma }}}|\rightarrow |X|$, it immediately follows that their restrictions are homotopic as maps $|{\varSigma }|\rightarrow |X|$ as well.

Further, assume that all maximal simplices have dimension *d*. Let $\tau \in {\varSigma }$ be a *d*-dimensional simplex and let $\tau ^{int}$ be the simplex in $\mathrm {Esd}_k(\tau )$ spanned by the vertices

$$\begin{aligned} (l+1,l,\ldots ,l), \ldots , (l,\ldots ,l,l+1), \end{aligned}$$

that is, the simplex in the interior of $\tau$ that is mapped by $pf'$ to ${\tilde{\sigma }}$. Let $H_\tau (\cdot , 1): |\tau |\rightarrow |\tau |$ be a linear map that takes $|\tau |$ linearly to $|\tau ^{int}|$ via mapping the *i*’th vertex to $(l,\ldots , l+1,1\ldots , l)$ where the $l+1$ is on position *i*. Further, let $H_{\tau }$ be a linear homotopy $|\tau |\times [0,1]\rightarrow |\tau |$ between the identity $H_\tau (\cdot , 0)=\mathrm {id}$ and $H_\tau (\cdot , 1)$. The composition $|p f'| H_{\tau }$ then gives a homotopy $|\tau |\times [0,1]\rightarrow |X|$ between the restrictions $(|pf'|)|_{|\tau |}$ and $(|f|)|_{|\tau |}$. For a general $x\in |{\varSigma }|$, there exists a maximal *d*-simplex $|\tau |$ such that $x\in |\tau |$ and we define a homotopy

$$\begin{aligned} (x,t)\,\mapsto |p f'| H_\tau (x,t). \end{aligned}$$

It remains to show that this map is independent on the choice of $\tau$.

Let us denote the (ordered) vertices of $\tau$ by $\{v_0,v_1,\ldots ,v_d\}$ and let $\delta \subseteq \tau$ be one of its faces: further, let $w_i$ be the vertex of $\tau ^{int}$ with barycentric coordinates $(l,\ldots ,l,l+1,l,\ldots ,l)/k$ in $|\tau |$ such that the $l+1$ is in position *i*. The homotopy $H_{\tau }$ sends points in $|\delta |$ onto the span of points $w_i$ for which $v_i\in \delta$. For $y\in |\delta |$, the *j*-th barycentric coordinate of $H_{\tau }(y,t)$ is equal to $t\,(l/k)$ for each $j\notin \delta$. In particular, the *j*-th coordinate of $H_{\tau }(y,t)$ is between 0 and *l* / *k* for $j\notin \delta$, and hence it is not the “dominant” coordinate. It follows that each $z:=H_{\tau }(x,t)$ is contained in the interior of a unique simplex ${\varDelta }$ of $\mathrm {Esd}_k(\tau )$ such that $v_{{{\mathrm{arg\,max}}}x}\in \delta$ for all vertices *x* of ${\varDelta }$.

Let $i_0< i_1\cdots < i_k$ be the indices with $v_{i_j}\in \delta$, and $j_1< \cdots < j_{d-k}$ be the remaining indices. Let $\tau '=(v_0',\ldots ,v_d')$ be another *d*-simplex containing $\delta$ as a face. Assume, for simplicity, that the vertices of $\tau '$ are ordered so that vertices of $\delta$ have orders $i_0,\ldots ,i_k$—such as it is in $\tau$. Let $\sigma ,\sigma '$ be the lift of $f(\tau )$, $f(\tau ')$ respectively, and $V_i$, $V_i'$ the *i*-th vertex of $\sigma$, $\sigma '$ respectively.

We define a “mirror” map $m: |\tau |\rightarrow |\tau '|$, which to a point with barycentric coordinates $(x_0,\ldots ,x_d)$ with respect to $\tau$ assigns a point in $|\tau '|$ with the same barycentric coordinates with respect to $\tau '$. Clearly, $H_{\tau '}(y,t)=m(H_{\tau }(y,t))$ for $y\in |\tau |$ and whenever *z* is in the interior of a simplex ${\varDelta }\in \mathrm {Esd}_k(\tau )$, then *m*(*z*) is in the interior of $m({\varDelta })$, where vertices of ${\varDelta }$ and $m({\varDelta })$ have the same barycentric coordinates with respect to $\tau$ and $\tau '$, respectively. If, moreover, ${\varDelta }$ is such that each of its vertices *r* have coordinates $\le l/k$ on positions $j_1,\ldots , j_{d-k}$, then $V_{{{\mathrm{arg\,max}}}r}=V_{{{\mathrm{arg\,max}}}m(r)}'$.

To summarize these properties, $H_{\tau }(y,t)$ and $H_{\tau '}(y,t)$ satisfy that^20^

- they have the same coordinates wrt. $\tau$, $\tau '$, respectively,
- they are in the interior of simplices ${\varDelta }\in \mathrm {Esd}_k(\tau )$, ${\varDelta }'\in \mathrm {Esd}_k(\tau ')$ whose vertices have the same coordinates wrt. $\tau$, $\tau '$, respectively,
- the ${{\mathrm{arg\,max}}}$ labeling induces the same labeling of vertices of ${\varDelta }$, ${\varDelta }'$ by vertices of $\delta$, respectively.

**Fig. 8:**
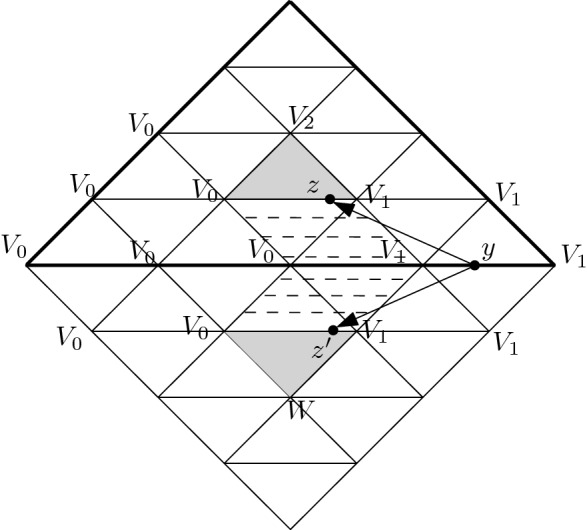
The homotopy $H_{\tau }$ takes *y* linearly into *z* and $H_{\tau '}$ takes *y* into $z'$. Due to the symmetry represented by the horizontal line, $|pf'|$ maps $H_{\tau }(y,t)$ into the same point of *X* as $|pf'| H_{\tau '}(y,t)$

The map $pf'$ takes each *m*-simplex ${\varDelta }$ in $\mathrm {Esd}_k(\tau )$ with vertices $t_u$ labeled by $V_{{{\mathrm{arg\,max}}}t_u}$ onto $p(V_{{{\mathrm{arg\,max}}}t_0},\ldots , V_{{{\mathrm{arg\,max}}}t_m})$ and it follows from the above properties that $m({\varDelta })$ is mapped to the same simplex. We conclude that $|pf'| H_{\tau }(y,t)=|pf'|H_{\tau '}(y,t)$ for each $y\in |\delta |$ and $t \in [0,1]$ (Fig. 8). $\square$

## Footnotes

1 This follows via a standard reduction from a result of Adyan (1955) and Rabin (1958) on the algorithmic unsolvability of the triviality problem of a group given in terms of generators and relations; we refer to the survey Soare (2004) for further background.

2 That is, they compute integers $r,q_1,\ldots ,q_k$ such that $\pi _d(X)$ is isomorphic to ${\mathbb {Z}}^r\oplus {\mathbb {Z}}_{q_1}\oplus \cdots \oplus {\mathbb {Z}}_{q_k}$.

3 Similarly, the algorithm in Čadek et al. (2014b) constructs an auxiliary chain complex *C* such that $\pi _d(X)$ is isomorphic to the homology group $H_{d+1}(C)$ and computes the latter.

4 Similarly as before, the algorithm in Čadek et al. (2014a) computes [*X*, *Y*] as the set [*X*, *P*] for some auxiliary space *P* (a stage of a *Postnikov system* for *Y*) and represents the elements of $[X,Y]\cong [X,P]$ as maps from *X* to *P*, but not as maps to *Y*.

5 The Whitehead tower can is a “dual” construction to the Postnikov tower, beginning with the space *X* and gradually eliminating the homotopy groups “’form the bottom”. For the readers familiar with Moore–Postnikov towers (or relative Postnikov towers), the Whitehead tower is the Moore–Postnikov tower for $* \rightarrow X$, see e.g. Goerss and Jardine (1999), Def. IV, 2.9.

6 A homotopy $F: {S}^k \times I \rightarrow X$ is pointed if $F(*,t) = x_0$ for all $t \in I$.

7 Formally, elements of *F*(*S*) are sequences of symbols $s^{\epsilon }$ for $\epsilon \in \{1,-1\}$ and $s\in S$ with the relation $s^1 s^{-1}=1$, where 1 represents the empty sequence. The group operation is concatenation.

8 We remark that the paper Čadek et al. (2014b) uses a different formalization of twitsed cartesian product than the one employed by us. However, the paper Filakovský (2012), on which the Corollary 3.18 of Čadek et al. (2014b) is based, can be reformulated in context of the definition used here. We do not provide full details, only remark that one has to make a choice of *Eilenberg–Zilber reduction data* that corresponds to the definition of twisted cartesian product.

9 A version of this lemma is given as Barratt (1956, Theorem 2) or Čadek et al. (2013b, Proposition 3.5). However, we also need the fact that $|Y^{sc}|$ is homeomorphic to |*Y*|, which is not explicitly mentioned in the references, but follows easily from the construction.

10 The subdivision *Sd*(*Y*) has geometric realization homeomorphic to |*Y*| by Fritsch and Piccinini (1990, Thm 4.6.4). The realization of *Sd*(*X*) is a regular CW complex and $B_*(Sd(Y))$ coincides with the first derived subdivision of this regular CW complex, as defined in Geoghegan (2007, p. 137). The geometric realization of the resulting simplicial complex is still homeomorphic to |*Y*| and |*Sd*(*Y*)| by Geoghegan (2007, Prop. 5.3.8).

11 The constructed map *f* does not necessarily preserves orientations: it only maps simplices to simplices.

12 With a slight abuse of language, we assume that each $X_k$ not only a simplicial set but also its algorithmic representation with a specified size such as explained in Sect. 3.

13 Kan uses a slightly different convention in Kan (1958a) but the resulting properties are the same. The sequence $\beta _0,\ldots ,\beta _k$ can be interpreted as a discrete path from $\alpha$ to the identity element.

14 For $t=\sum _j k_j {\overline{\sigma }}_j$, we may choose $h=\prod _j {\overline{\sigma }}_j^{k_j}$ (choosing any order of the simplices).

15 The connectivity assumption on *F* was exploited in the existence of the contraction $c_j^A$ on the Abelian part.

16 By nontrivial we mean that $\gamma \ne 1_x$ for any $x\in X_{d-1}$.

17 For example, $[a,1] [b,2] [b,2]^{-1} [a,1]^{-1}$ can be split into a sequence $([b,2], [b,2]^{-1}), ([a,1], [a,1]^{-1})$.

18 If $\dim (\tau )=d$ is maximal, then $j=l$ and this most-middle simplex has particularly nice coordinates $(l+1,l,\ldots , l), \ldots , (l,\ldots , l,l+1)$.

19 Here the assumption on the fixed dimension *d* is crucial.

20 In general, vertices of $\delta$ may have different order in $\tau$ and $\tau '$ and the assumption on compatible ordering was chosen only to increase readability. If $i_0'< \cdots <i_{k}'$ are such that $v_{i_j'}'=v_{i_j}$ (orders of $\delta$-vertices wrt. $\tau '$) and $j_1'<\cdots <j_{d-k}'$ are positions of the remaining vertices in $\tau '$, then *m* is defined so that it maps $x\in |\tau |$ with $\tau$-coordinates $(x_0,\ldots , x_d)$ into $x'\in |\tau '|$ with coordinates $x_{i_j'}'=x_{i_j}$ and $x_{j_k}'=x_{j_k}$.
